# Comparative transcriptome analysis of purple-fleshed sweet potato and its yellow-fleshed mutant provides insight into the transcription factors involved in anthocyanin biosynthesis in tuberous root

**DOI:** 10.3389/fpls.2022.924379

**Published:** 2022-08-08

**Authors:** Wen Dong, Linfei Tang, Yali Peng, Yuzhi Qin, Yuan Lin, Xingyao Xiong, Xinxi Hu

**Affiliations:** ^1^Hunan Provincial Engineering Research Center for Potatoes, College of Horticulture, Hunan Agricultural University, Changsha, China; ^2^Agricultural Genomics Institute at Shenzhen, Chinese Academy of Agricultural Sciences, Shenzhen, China; ^3^Key Laboratory for Vegetable Biology of Hunan Province, Changsha, China

**Keywords:** sweet potato, tuberous root, anthocyanins, transcription factor, transcriptome analysis

## Abstract

In various plant species, many transcription factors (TFs), such as MYB, bHLH, and WD40, have been identified as regulators of anthocyanin biosynthesis in underground organs. However, the regulatory elements of anthocyanin biosynthesis in the tuberous roots of sweet potato have not been elucidated yet. Here, we selected the purple-fleshed sweet potato cultivar “Zhezi1” (ZZ*^P^*) and its spontaneous yellow-fleshed mutant “Xinli” (XL*^Y^*) to investigate the regulatory mechanism of the anthocyanin biosynthesis in the tuberous roots of sweet potato. By analyzing the *IbMYB1* genotype in ZZ*^P^* and XL*^Y^*, we found that the *IbMYB1-2*, a MYB TF involved in anthocyanin biosynthesis, was missing in the XL*^Y^* genome, which might lead to an extreme decrease in anthocyanins in XL*^Y^*. A comparative transcriptome analysis of ZZ*^P^* and XL*^Y^* was conducted to find the TFs involved in anthocyanin biosynthesis in ZZ*^P^* and XL*^Y^*. The anthocyanin structural genes were significantly enriched among the differentially expressed genes. Moreover, one MYB activator (*IbMYB1*), one bHLH (*IbbHLH2*), three WRKY activator candidates (*IbWRKY21*, *IbWRKY24*, and *IbWRKY44*), and two MYB repressors (*IbMYB27* and *IbMYBx-ZZ*) were highly expressed in ZZ*^P^* accompanied with anthocyanin structural genes. We also tested the expression of these TFs in six purple- and two orange-fleshed sweet potato cultivars. Interestingly, most of these TFs were significantly positively correlated with anthocyanin contents in these cultivars. The function of the anthocyanin biosynthesis repression of *IbMYB27* and *IbMYBx-ZZ* was verified through transient co-transformation with *IbMYB1* into tobacco leaves. Further functional verification of the above TFs was conducted by Y2H, BiFC, and dual-luciferase assays. These tests showed that the MYB-bHLH-WD40/MYB-bHLH-WD40-WRKY complex activated the promoter of anthocyanin structural gene *IbDFR* and promoters for *IbWRKY44*, *IbMYB27*, and *IbMYBx-ZZ*, indicating reinforcement and feedback regulation to maintain the level of anthocyanin accumulation in the tuberous roots of purple-fleshed sweet potato. These results may provide new insights into the regulatory mechanism of anthocyanin biosynthesis and accumulation in underground organs of sweet potatoes.

## Introduction

Sweet potato (*Ipomoea batatas* [L.] Lam.) is the seventh-most important food crop in the world after wheat, rice, maize, potato, barley, and cassava. The global production of sweet potato is centered in Asia, and China is the largest producer, accounting for 56.05% of the total production (FAOSTAT, 2020). Sweet potatoes are rich in carbohydrates, dietary fibers, β-carotene, minerals, and other nutrients (Endrias et al., 2016). Moreover, cultivars with purple flesh are rich in anthocyanins (Mano et al., 2007).

In plants, structural genes encoding phenylalanine ammonia lyase (*PAL*), chalcone synthase (*CHS*), chalcone isomerase (*CHI*), flavanone 3-hydroxylase (*F3H*), flavonoid 3′-hydroxylase (*F3’H*), dihydroflavonol 4-reductase (*DFR*), anthocyanin synthase (*ANS*), and glutathione S-transferase (*GST*) are involved in anthocyanin biosynthesis (Holton and Cornish, 1995). These anthocyanin biosynthesis genes are activated by a transcriptional activation complex comprising basic helix–loop–helix (bHLH), R2R3-MYB, and WD-repeat (WDR) proteins (Chu et al., 2013; Albert et al., 2014). The R2R3-MYB factors in the complex are most crucial for the spatial and temporal localization of anthocyanins (Davies et al., 2012). For example, in the overground part, AtPAP1, AtMYB113, and AtMYB114 in *Arabidopsis* (Gonzalez et al., 2010) and PhAN2 in petunia (Quattrocchio et al., 1999) have been reported. In underground organs, DcMYB7 and DcMYB113 in carrot (Xu et al., 2019; Bæksted Holme et al., 2021) and AN1 in potato have been identified (Liu et al., 2016). However, two types of MYB repressors, i.e., R2R3-MYBs and R3-MYBs, which repress anthocyanin biosynthesis, are identified. For example, FaMYB1 (R2R3-MYB) in strawberry (Aharoni et al., 2001; Salvatierra et al., 2013), PhMYB27 (R2R3-MYB), and PhMYBx (R3-MYB) in petunia (Albert et al., 2011) have been reported. The MYB-bHLH-WD40 (MBW) activation complex and MYB repressors participate in anthocyanin biosynthesis in petunia leaf through hierarchical and feedback regulation to prevent inappropriate anthocyanin level (Albert et al., 2014).

Moreover, WRKY can promote anthocyanin biosynthesis by forming MYB-bHLH-WD40-WRKY (MBWW) complexes with MYB, bHLH, and WD40 (Verweij et al., 2016). In *Arabidopsis thaliana*, AtTTG2 (WRKY) can interact with AtTTG1 (WD40) to activate the accumulation of PAs in the seed coat (Gonzalez et al., 2016). In *Petunia hybrida*, PhPH3 can directly interact with the MBW complex to regulate the biosynthesis of anthocyanins (Verweij et al., 2016). WRKY proteins, as a large family of transcription factors (TFs) in plants, influence physiological processes, such as plant stress resistance, growth, and development (Alessandra et al., 2016). On the basis of the number of WRKY domains (WRKYGQ) and the characteristics of the zinc finger’s structure, WRKY proteins can be divided into three groups (Eulgem et al., 2000). However, reports on the regulation of anthocyanin biosynthesis by WRKY TFs in sweet potatoes are few.

Previous research suggested that anthocyanin biosynthesis in the storage roots of purple-fleshed sweet potato is regulated by an MYB activator, i.e., IbMYB1 (Mano et al., 2007). Two genotypes of the *IbMYB1* gene, named *IbMYB1-1* and *IbMYB1-2*, are found in the genome of a purple-fleshed sweet potato cultivar Ayamurasaki, whereas its spontaneous white-fleshed mutant and other white- or yellow-fleshed cultivars have only *IbMYB1-1* (Tanaka et al., 2012). Although *IbMYB1-1* and *IbMYB1-2* have identical coding sequences, only *IbMYB1-2* is identified to be responsible for anthocyanin accumulation in tuberous roots (Tanaka et al., 2012). In sweet potato, the molecular mechanism for the loss of anthocyanins in red young leaves gradually turning green is explored (Deng et al., 2020). Three MYB activator genes (i.e., *IbMYB1*, *IbMYB2*, and *IbMYB3*) are simultaneously expressed with four MYB repressor genes (i.e., *IbMYB27*, *IbMYB4a*, *IbMYB4c*, and *IbMYBx*) in the early stage of leaf development, and these MYB TFs are involved in the hierarchical and feedback regulation of anthocyanin accumulation (Deng et al., 2020). However, as underground organs, the tuberous roots of purple-fleshed sweet potato remain purple during the whole development stage. Therefore, the pattern of spatial and temporal localization of anthocyanins in underground organs may be different. Whether MYB repressors, MYB activators, and WRKYs collaboratively participate in the regulation of anthocyanin biosynthesis in underground organs remains to be clarified.

In this manuscript, a spontaneous yellow-fleshed mutant Xinli (XL*^Y^*) is obtained from the purple-fleshed sweet potato cultivar “Zhezi1” (ZZ*^P^*). The transcriptome analysis of two homologous materials ZZ*^P^* and XL*^Y^* is performed to elucidate the regulatory mechanism of the anthocyanin biosynthesis in the tuberous roots of purple-fleshed sweet potato. Candidate TFs are cloned, and their functions are verified. Our results suggest that MYB activators, bHLHs, WRKYs, and MYB repressors are collaboratively involved in the regulation of anthocyanin biosynthesis in the tuberous roots of purple-fleshed sweet potato to prevent the excessive accumulation of anthocyanins.

## Materials and methods

### Plant materials

A spontaneous yellow-fleshed mutant of ZZ*^P^* was obtained from the experimental field of Hunan Agricultural University in 2012 and was named XL*^Y^*. Eight recent Chinese sweet potato cultivars with different genetic backgrounds, including six purple- (i.e., XZ118, GZ11, GZ10, YZ7, NY76, and XZ7) and two orange-fleshed (i.e., X19 and G87) sweet potatoes, bred in different departments in different regions of China were grown in an experimental field of the Hunan Agricultural University in Changsha, Hunan in the autumn of 2019. Each sample replicate consisted of five tubers from different plants in the tuber mature stage, and three biological replicates were used. Cylinders with diameter of 1 cm from the middle parts of tuberous roots were stored in liquid nitrogen for transcriptome analysis and quantitative real-time (qRT)-PCR. Others were used in determining the content of anthocyanins. Tobacco (*Nicotiana tabacum* L., for transient assays and *Nicotiana benthamiana* L. for dual-luciferase assays) was grown in a pot with 16 h/8 h light/dark photocycle at 25°C.

### Extraction and quantification of anthocyanins

Anthocyanins in tuberous roots of sweet potato were extracted and quantified in accordance with the methods described by Sun and Guo (2008). In brief, 0.5 g tuber tissues were immersed and extracted with methanol and 0.1% HCl. Anthocyanin levels from methanol extracts were estimated in accordance with equation: *A* = ([A520 × 50]/[98.2 × 0.5]) × 100. Absorbance was measured using the Multiskan spectrum device (Thermo Scientific Multiskan GO 1510, Finland).

### RNA and DNA isolation and genomic PCR of ZZ*^P^* and XL*^Y^*

The total RNAs of experimental materials used in this paper were extracted using the RNA-prep Pure Plant Plus Kit (Polysaccharides and Polyphenolics-rich; Tiangen Biotech [Beijing] CO. LTD.). The RNA of each sample was reverse-transcribed into cDNA by the premix kit (Tiangen Biotech [Beijing] CO. LTD.). The total DNAs of experimental materials used in this manuscript were isolated using a plant genomic DNA kit (Tian gen, Beijing, China). Genomic PCR was conducted for *IbMYB1* and structural genes using the PCR conditions and primers described by Mano et al. (2007). For the analysis of the *IbMYB1* genotype in ZZ*^P^* and XL*^Y^*, *IbMYB1-1* and *IbMYB1-2* fragments were amplified using PCR conditions and primers (Supplementary Table 1) described by Tanaka et al. (2012).

### Transcriptome analysis

Approximately 3 μg RNA per sample was used as input material for RNA sample preparations. The NEB Next^®^ Ultra RNA library prep kit for Illumina^®^ (NEB, MA, United States) was used to generate sequencing libraries. Index codes were added to attribute sequences to each sample.

A qualified complete cDNA library was obtained using the PacBio sequel platform and sequenced in a cell. The Agilent Bioanalyzer2100 sequencing platform (Illumina) was used to sequence second-generation libraries. Following the manufacturer’s instructions, the clustering of index-coded samples was performed on a cBot cluster generation system with the TruSeq PE cluster kit v3-cBot-HS (Illumina). Briefly, mRNA was enriched by Oligo (dT) beads. The enriched mRNA was fragmented into short fragments with fragmentation buffer and reverse-transcribed into cDNA with random primers. The second-strand cDNA was synthesized by DNA polymerase I, RNase H, dNTP, and buffer. Then, cDNA fragments were purified, end repaired, poly (A) added, and ligated to Illumina sequencing adapters. Ligation products were selected by size through agarose gel electrophoresis, PCR amplified, and sequenced using the AMPure XP system. High-quality clean reads were first aligned to *de novo* cognate assemblies of “ZZ*^P^*” and “XL*^Y^*” and mapped to the reference genome by the HISAT v2.1.0 after the release of the genomic sequence of the sweet potato cultivar “Taizhong 6” (Yang et al., 2017). No fundamental difference was displayed in the comprehensive analysis of the categories of expressed genes. However, subsequent analysis with the reference genome for specific gene sequences showed considerable incompleteness and inaccuracy that hindered further investigation. Alignment to *de novo* cognate assemblies was used as the main data to bypass these issues, whereas alignment to the “Taizhong 6” reference genome was used as auxiliary data for the subsequent homologous cloning of the promoters together with genomes of *I. nil* (Hoshino et al., 2016), *I. trifida*, and *I. triloba* (Wu et al., 2018). Differential expression gene analysis was performed on ZZ*^P^* and XL*^Y^* with the DESeq2 R package (1.22.2). *q*-value was < 0.05. For the anthocyanin biosynthesis genes, differentially expressed genes (DEGs) were recruited by log_2_ (fold change) > 2, and the *q*-value was < 0.01. Unigenes were functionally annotated by searching against NCBI nucleotide sequences (Nt), NCBI non-redundant protein, Gene Ontology (GO), Kyoto Encyclopedia of Genes and Genomes (KEGG), euKaryotic Ortholog Groups, Protein family, and Swiss-Prot databases, and the *E*-value was 10^–5^.

### qRT-PCR

The primers used for qRT-PCR analysis were designed using the Primer 5 (Supplementary Table 1). qRT-PCR was performed using the Roche LightCycler^®^ 480II system with the TSINKE2 × T5 Fast qPCR Mix (SYBR Green I) (TSINKE, China) under the following conditions: 95°C for 60 s, 40 cycles of 95°C for 10 s, and 60°C for 15 s. The expression levels of genes were normalized to the level of constitutive *IbACTIN* expression. The 2^–ΔCT^ method was used in analyzing the qRT-PCR results for eight varieties of sweet potato and tobacco leaves, whereas the 2^–^^ΔΔCT^ method was used for ZZ*^P^* and XL*^Y^*.

### Phylogenetic analysis for the anthocyanin-related WRKY and MYB

Phylogenetic trees were constructed by MEGA X (Tamura et al., 2011). Parameters for the NJ tree were set as p-distance model and pairwise deletion with the bootstrap value as 1,000. According to results, the TF clusters of MYB and WRKY were used in protein sequence alignment analysis through the GenDoc.

### Gene function assays

For the gene function analysis, the full-length coding sequences of *IbMYB1*, *IbMYB27*, and *IbMYBx-ZZ* were cloned using the Planta Super-Fidelity DNA Polymerase (Abm, BC, Canada) and inserted into a p2300 vector under the control of a 35S promoter. Primer sequences are listed in Supplementary Table 1. The transformation of recombinant vectors into GV3101 strains of *Agrobacterium tumefaciens* was performed in accordance with the methods described by Zhou et al. (2019).

*Agrobacterium* cells harboring *IbMYB1*, *IbMYBx-ZZ*, *IbMYB27*, and empty vector (CK) were mixed in different ratios and used in testing the repressing effects of IbMYB27 and IbMYBx-ZZ. The methods of mixing *Agrobacterium* cells and transforming into *N. tabacum* leaves were performed in accordance with the methods described by Zhou et al. (2019). Plants were incubated in the dark for 24 h and moved to a greenhouse with artificial irradiance (16 h day). Photographs were taken 7 days after injection, and the total RNA was collected for extraction when necessary.

### Yeast two-hybrid assays

The open reading frames (ORFs) of *IbMYB1*, *IbMYB27*, *IbMYBx-ZZ*, *IbWRKY44*, and *IbWD40* were cloned into the pGADT7 plasmid to generate IbMYB1-AD, IbMYB27-AD, IbMYBx-ZZ-AD, IbWRKY44-AD, and IbWD40-AD, respectively. The ORFs of *IbMYB1*, *IbbHLH1*, *IbbHLH2*, *IbWD40*, and *IbMYB27* were inserted into the pGBKT7 plasmid to generate IbMYB1-BD, IbbHLH1-BD, IbbHLH2-BD, IbWD40-BD, and IbMYB27-BD, respectively. The primers used to clone these genes are listed in Supplementary Table 1. Recombinant vectors were transformed into Y187 yeast (Clontech, CA, United States) by PEG3350-mediated transformation. The transformed yeast cells were cultured in -T/-L (-tryptophan/-leucine) and -T/-L/-H/-A (-tryptophan/-leucine/-histidine/-adenine) media for 3 days to observe yeast growth.

### Bimolecular fluorescence complementation assays

The ORFs of *IbMYB1*, *IbMYB27*, *IbMYBx-ZZ*, *IbbHLH1*, *IbbHLH2*, *IbWD40*, and *IbWRKY44* were inserted into pSPYNE-35S/pUC-SPYNE (provided by our own laboratory) or pSPYCE-35S/pUC-SPYCE plasmids to generate IbbHLH1-YFPC, IbbHLH2-YFPC, IbWD40-YFPC and IbMYB1-YFPN, IbWRKY44-YFPN, IbMYB27-YFPN, IbMYBx-ZZ-YFPN, respectively. Recombinant vectors were transformed into GV3101 strains of *Agrobacterium tumefaciens* in accordance with the methods described in Section “Gene function assays.” *A. tumefaciens* mixtures (ratio = 1:1) were infiltrated into *N. benthamiana* leaves and cultured in a greenhouse for 2 days before sample collection. Fluorescence was detected using a confocal laser scanning microscope (TCIT SP2, Leica).

### Dual-luciferase assays

The ORFs of IbMYB1, IbbHLH1, IbbHLH2, IbWD40, IbWRKY44, IbMYBx-ZZ, and IbMYB27 were cloned into the pGreen62-SK plasmid (provided by our own laboratory) to generate IbMYB1-pGreen62-SK, IbbHLH1-pGreen62-SK, IbbHLH2-pGreen62-SK, IbWD40-pGreen62-SK, IbWRKY44-pGreen62-SK, IbMYBx-ZZ-pGreen62-SK, and IbMYB27-pGreen62-SK, respectively. The promoter sequences of IbDFR, IbMYB27, IbMYBx-ZZ, and IbWRKY44 were inserted into the pGreen0800-LUC plasmid to generate pIbDFR-LUC, pIbMYB27-LUC, pIbMYBx-ZZ-LUC, and pIbWRKY44-LUC, respectively. The primers used to clone these gene promoters are listed in Supplementary Table 1. Recombinant vectors were transformed into the GV3101 strains of *A. tumefaciens* in accordance with the methods described in Section “Gene function assays.” *A. tumefaciens* mixtures (ratio = 1:1) were infiltrated into *N. benthamiana* leaves and cultured in a greenhouse for 2 days before sample collection. Afterward, 0.2 mM luciferin (Biyuntian, Jiangsu, China) was infiltrated into the same positions where *A. tumefaciens* was infiltrated and held in a dark room for 0.5 h. The luciferase activity was detected by live plant imaging system (Xenogen, Alameda, CA, United States, IVIS Spectrum). The LUC imaging was visualized using the Tanon gel imaging software. Promoter sequences are presented in Supplementary Table 3.

### Statistical analysis

Samples were subjected to statistical analysis by using the IBM SPSS statistical software.^1^ Data were analyzed by one-way analysis of variance (ANOVA), and the *t*-test (*P* < 0.01) was used to separate the means in ZZ*^P^* and XL*^Y^*.

## Results

### Phenotypes of the ZZ*^P^* and XL*^Y^* mutant

**FIGURE 1:**
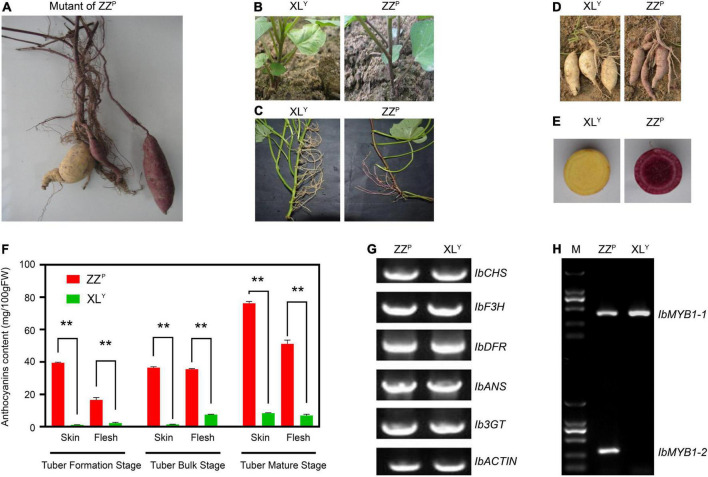
Phenotype and genotype of the Zhezi.1 (ZZ*^P^*) and Xinli (XL*^Y^*) mutant. **(A)** First appearance of ZZ*^P^* mutant. **(B)** Plants of XL*^Y^* (Left) and ZZ*^P^* (Right) grown from tuberous roots. **(C)** Stems, **(D)** tuberous roots, and **(E)** cross-section of tuberous roots of XL*^Y^* (Left) and ZZ*^P^* (Right). **(F)** Anthocyanin content in tuberous roots of ZZ*^P^* and XL*^Y^* in different parts and stages. Error bars indicate the standard deviation from three biological replicates. Asterisks represent statistically significant differences (**P < 0.01), analyzed using Student’s *t*-test. **(G)** Genomic PCR for full CDS of structural genes involved in anthocyanin biosynthesis on ZZ*^P^* and XL*^Y^*. **(H)** Genotype of *IbMYB1* in ZZ*^P^* and XL*^Y^* mutant. M, Marker of 2000.

XL*^Y^*, a spontaneous tuberous root mutant of ZZ*^P^*, was first found among the ZZ*^P^* tuberous root when it was harvested in the field (Figure 1A). The skin color of the storage roots of XL*^Y^* became white. The pigmentation on the stem of the plant, which first grew from the tuberous roots of the XL*^Y^*, was slighter than that of ZZ*^P^* (Figure 1B). No anthocyanin pigmentation was observed in stems, aerial roots, fiber roots, and storage roots of XL*^Y^* plants growing from stem cuttings (Figures 1C–E). The anthocyanin contents in the skin and flesh of the storage roots of XL*^Y^* were significantly (*p* < 0.01) reduced to about 1/9th and 1/7th, respectively, compared with those of ZZ*^P^* in the tuber mature stage (Figure 1F). However, the color of the leaves seemed unchanged (Figures 1B,C). No other evident phenotypic difference was observed on plants between XL*^Y^* and ZZ*^P^* except for anthocyanin accumulation.

### Analysis of the *IbMYB1* genotype in ZZ*^P^* and XL*^Y^* mutant

Genomic PCR was conducted for structural gene coding sequence in accordance with the method described by previous research (Mano et al., 2007) to explore whether gene deletion in XL*^Y^* resulted in a remarkable decrease in anthocyanin accumulation. All tested genes, including *IbCHS*, *IbF3H*, *IbDFR*, *IbANS*, and *Ib3GT*, were detected in ZZ*^P^* and XL*^Y^* (Figure 1G). Results implied that there was no missing of these genes in ZZ*^P^* and XL*^Y^*.

Previous research suggested that two variants of *IbMYB1*, namely, *IbMYB1-1* and *IbMYB1-2*, are found in the genomes of purple-fleshed cultivar Ayamurasaki and that *IbMYB1-2* is lost in its white-fleshed mutant cultivar AYM96 (Tanaka et al., 2012). The identification of the *IbMYB1* genotype was conducted on ZZ*^P^* and XL*^Y^* in accordance with a previously described method (Tanaka et al., 2012). The *IbMYB1-1* variant was detected in ZZ*^P^* and XL*^Y^*, but the *IbMYB1-2* variant was only detected in ZZ*^P^* (Figure 1H). These results implied that the missing *IbMYB1-2* in XL*^Y^* might lead to remarkable downregulation of *IbMYB1*, eventually leading to a dramatic reduction in anthocyanin accumulation in the XL*^Y^* mutant.

### Transcriptome analyses of ZZ*^P^* and XL*^Y^* mutant

**FIGURE 2:**
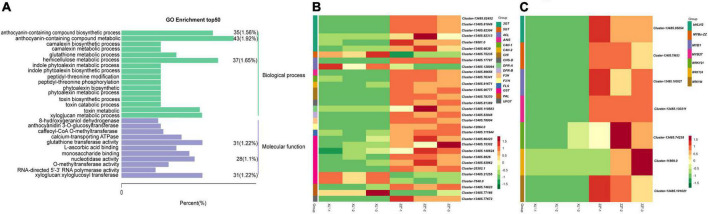
Differentially expressed genes (DEGs) in tuberous roots of ZZ*^P^* and XL*^Y^*. **(A)** Top 50 pathways with the most significant enrichment in GO term between ZZ*^P^* and XL*^Y^*. The X-axis indicates the number and percentage of DEGs under each functional classification, whereas the Y-axis represents the enriched GO functional classification, which is divided into two categories: biological process and molecular function. **(B)** Heat map of differentially expressed structural genes relative to anthocyanin biosynthesis pathway between ZZ*^P^* and XL*^Y^*. **(C)** Heat map of differentially expressed TFs relative to the anthocyanin biosynthesis pathway between ZZ*^P^* and XL*^Y^*. Each row represents a gene, and each column represents a sample (XL*^Y^*-1, XL*^Y^*-2, and XL*^Y^*-3 represent three replicates of XL*^Y^* sample. ZZ*^P^*-1, ZZ*^P^*-2, and ZZ*^P^*-3 represent three replicates of ZZ*^P^* sample). Different colors in the clustering heat map represent the different expression levels of genes. Red and green colors indicate high and low expression levels, respectively.

RNA-seq was performed on tuberous roots of ZZ*^P^* and XL*^Y^* to screen DEGs in sweet potato tubers. The sequencing and assembly results implied the reliability of unigenes data (Supplementary Table 2). A total of 49.87 and 50.33 million raw reads were obtained, and the average Q30 of the raw reads were 91.09 and 91.17% in ZZ*^P^* and XL*^Y^*, respectively (Supplementary Table 2). A total of 3,876 DEGs, including 1,792 downregulated and 2,084 upregulated genes, were identified in XL*^Y^* compared with ZZ*^P^* (Supplementary Figure 1A). In the GO term, DEGs were further classified into 3,288 functional subcategories. In the “biological process” category, the “anthocyanin-containing compound metabolic process” (43 unigenes, 1.92%), “hemicellulose metabolic process” (37 unigenes, 1.65%), and “anthocyanin-containing compound biosynthetic process” (35 unigenes, 1.56%) were the most common assignments (Figure 2A). DEGs were assigned to 128 KEGG pathways. The “metabolic pathways,” “biosynthesis of secondary metabolites,” “phenylpropanoid biosynthesis,” and “flavonoid biosynthesis” categories were the top four enriched processes in DEGs (Supplementary Figure 1B). Hence, these results showed that the downregulated genes involved in flavonoid and anthocyanin biosyntheses might be the cause of the dramatic reduction in anthocyanin accumulation in XL*^Y^* mutant.

### Identification of the structural genes and transcription factors involved in flavonoid and anthocyanin biosyntheses in ZZ*^P^*

We focused on the structural genes and TFs involved in anthocyanin biosynthesis. The DEGs involved in the biosyntheses of anthocyanins and flavonoids were screened (Figures 2B,C). Among the differentially expressed structural genes involved in the biosyntheses of flavonoids and anthocyanins, 1 *4CL*, 1 *CHS*, 2 *CHI*, 1 *F3’H*, 1 *F3H*, 2 *DFR*, 1 *ANS*, 6 *3GT*, 1 *UFGT*, 1 *PAL*, 2 *C4H*, 1 *FLS*, and 6 *GST* genes in XL*^Y^* were significantly downregulated compared with those in ZZ*^P^* (Figure 2B). These results were consistent with the remarkable differences in anthocyanin accumulation in the storage roots of ZZ*^P^* and XL*^Y^*. Moreover, 260 TF genes, including 127 downregulated and 133 upregulated genes, were determined in XL*^Y^*. Among these TF genes, 3 MYB genes, i.e., *IbMYB1* (Cluster-13485.100527), *IbMYB27* (Cluster-13485.130311), *IbMYBx-ZZ* (Cluster-13485.79633); 3 WRKY genes, i.e., *IbWRKY21* (Cluster-13485.74238), *IbWRKY44* (Cluster-13485.101021), *IbWRKY24* (Cluster-11869.0); and 1 bHLH gene *IbbHLH2* (Cluster-13485.95054) in XL*^Y^* were markedly downregulated compared with those in ZZ*^P^* (Figure 2C).

**FIGURE 3:**
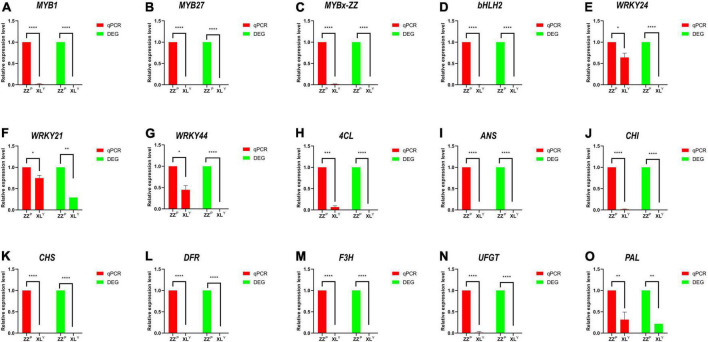
Verification of the relative expression level of DEGs in the tuberous roots of sweet potato by qRT-PCR. Expression levels were standardized to *GADPH*, and results in the ZZ*^P^* were set to 1. Expression patterns of 15 DEGs related to anthocyanin biosynthesis pathway including **(A–G)** TFs and **(H–O)** structural genes. The red bar shows relative gene expression level measured by qRT-PCR, and the green bar shows the FPKM of relative gene in RNA-seq. Error bars indicate the standard deviation from three biological replicates. Asterisks represent statistically significant differences (****P < 0.0001, ***P < 0.001, **P < 0.01, *P < 0.05), as analyzed using Student’s *t*-test.

RNA-seq data were further confirmed by selecting 15 related anthocyanin biosynthesis genes for RT-qPCR (Figure 3). Results indicated that the expression patterns from qRT-PCR assay correlated well with sequencing results.

### Functional verification of activator IbWRKY44 and two repressors IbMYBx-ZZ and IbMYB27

**FIGURE 4:**
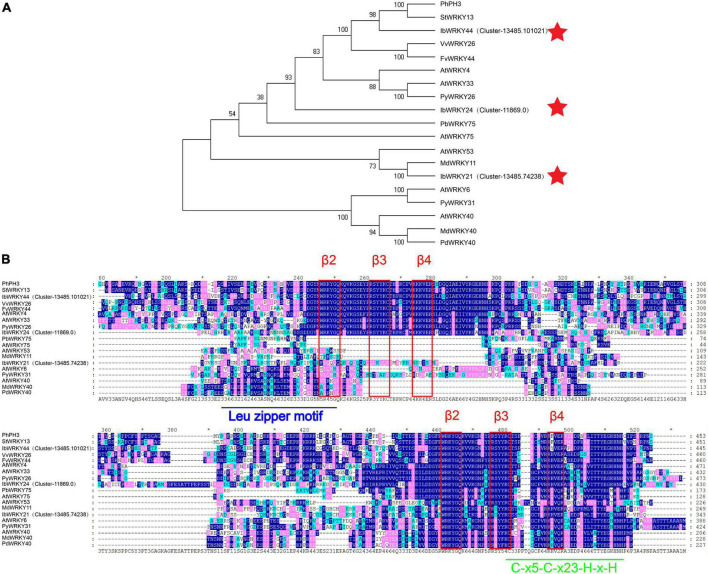
Phylogenetic analysis and protein sequence alignment for the putative WRKYs with function-verified homologous genes of other plant species. **(A)** Phylogenetic relationship of putative WRKYs in sweet potato and function-verified anthocyanin-related WRKYs in other species. Pentagram represents WRKYs screened from RNA-seq. **(B)** Protein sequence alignment for the putative MYBs with function-verified homologous genes of other plant species. Blue and green lines represent the Leu zipper motif and C-x_5_-C-x_23_-H-x-H motif, respectively. The red rectangle represents the β2-β3-β4 domains.

A phylogenetic tree of the WRKY family was constructed using the method described by Li et al. (2020). Phylogenetic analysis results indicated that the protein sequences of *IbWRKY21*, *IbWRKY24*, and *IbWRKY44* were homologous to those of anthocyanin-related genes, including *MdWRKY11*, *PhPH3*, *PbWRKY75*, *StWRKY13*, *VvWRKY26*, and *AtWRKY53* (Figure 4A). The derived polypeptide alignment of *IbWRKY24*, *IbWRKY44*, and *IbWRKY21* was similar to that of other WRKY TFs involved in anthocyanin biosynthesis, and all of these WRKY TFs contained WRKY (β2-β3-β4) and zinc-finger-like (C-x5-C-x23-H-x-H) motifs (Figure 4B). *IbWRKY44* and *IbWRKY24* had two WRKY motifs, and *IbWRKY21* had only one WRKY motif. However, the Leu zipper motif, which existed in MdWRKY40 and AtWRKY40 and consisted of a series of modules of seven residues, was not identified in *IbWRKY24*, *IbWRKY44*, and *IbWRKY21*.

Three MYB genes screened by RNA-seq were aligned with classic anthocyanin MYBs in other plants, such as *Arabidopsis*, *Petunia hybrida*, *Malus domestica*, *Fragaria* × *ananassa Duch*, *I. purpurea*, and *I. nil* for the prediction of their functions (Supplementary Figure 2A). *IbMYB1* and *IbMYB27* were named after their homologous genes in *I. batatas* and clustered into MYB activator members and FaMYB1-like repressor members, respectively (Supplementary Figure 2A). *IbMYBx-ZZ*, which encoded a protein of 80 amino acids, had an identity of 78% and 60% with *IbMYBx* and *PhMYBx*, respectively, by protein blast and had been identified to repress anthocyanin biosynthesis in sweet potato and petunia (Albert et al., 2011; Deng et al., 2020; Supplementary Figure 2B). *IbMYBx-ZZ* was clustered into PhMYBx-like R3-MYB members (Supplementary Figure 2A).

**FIGURE 5:**
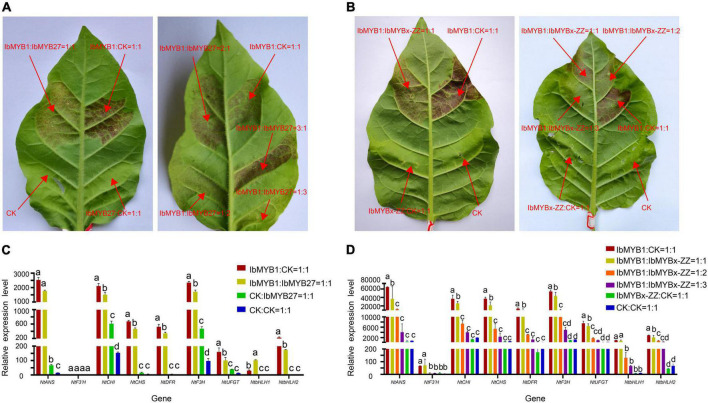
Functional analysis of the sweet potato IbMYB27 and IbMYBx-ZZ TFs. **(A,B)** Cotransformation of IbMYB1/IbMYB27 and IbMYB1/IbMYBx-ZZ-induced anthocyanin biosynthesis, as shown by transient expression assays in tobacco leaves. Tobacco leaves appeared 5 days after injection. Different ratios of IbMYB1, IbMYBx-ZZ, IbMYB27, or CK (empty vector) cotransformed into tobacco leaves. **(C,D)** Expression patterns of nine DEGs related to the anthocyanin biosynthesis pathway, including TFs and structural genes, after IbMYB1 and IbMYB27 or CK (empty vector) cotransformation into tobacco leaves. Leaf samples in panels **(C,D)** were cut out from the transformation part of panels **(A,B)**. The X-axis represents the gene, and the Y-axis represents the relative expression level of the gene. Error bars indicate the standard deviation from three biological replicates. Statistical significance was determined by one-way ANOVA (*P*-values reported). Significant differences between means (Duncan, *P* = 0.05) were indicated by different letters above the bar.

**FIGURE 6:**
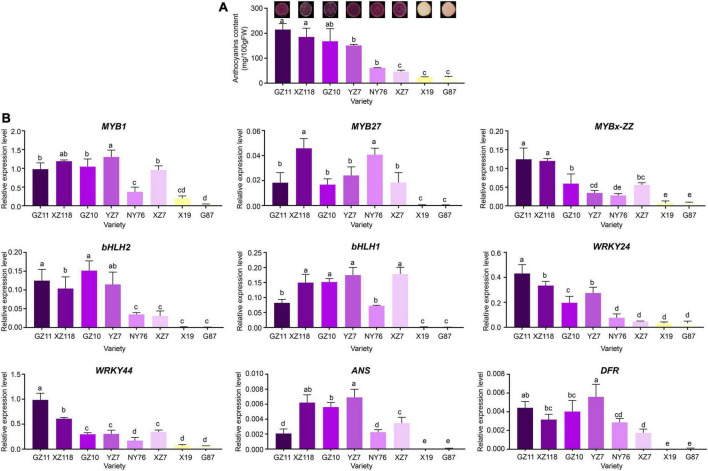
Total anthocyanin concentration and relative expression levels of key genes in various sweet potato accessions. **(A)** Cross-section of mature tuberous roots flesh and anthocyanin contents of the eight cultivars. The X-axis represents the cultivars, and the Y-axis represents the contents of anthocyanins. **(B)** Relative expression level of TFs and structural genes in the tuberous roots of the eight cultivars. The X-axis represents the gene, and the Y-axis represents the relative expression level of the gene. Error bars indicate the standard deviation from three biological replicates. Statistical significance was determined by one-way ANOVA (*P*-values reported). Significant differences between means (Duncan, *P* < 0.05) were indicated by different letters above the bar.

To elucidate the repression functions of IbMYBx-ZZ and IbMYB27 in anthocyanin biosynthesis, we induced transient transformation in tobacco leaves in accordance with the method described by Liu et al. (2016). *Agrobacterium* cells harboring IbMYB1, IbMYBx-ZZ, IbMYB27, and empty vector (CK) were mixed in different ratios and coinfiltrated into tobacco leaves. As shown in Figure 6, the purple pigment was observed on the tobacco leaves when IbMYB1 was transformed in tobacco leaves, whereas no purple pigment was observed on patches when IbMYB27 or IbMYBx-ZZ was transformed alone in tobacco leaves. The lighter purple pigment was observed on leaves when IbMYB1 was cotransformed with IbMYB27 or IbMYBx-ZZ (Figures 5A,B). Moreover, qRT-PCR results showed that the expression levels of TFs and structural genes in tobacco leaves were significantly downregulated when IbMYB1 was cotransformed with IbMYB27 and IbMYBx-ZZ (Figures 5C,D). These results indicated that IbMYB27 and IbMYBx-ZZ could repress anthocyanin biosynthesis, which was induced by IbMYB1.

### Expression levels of *IbMYBs*, *IbbHLHs*, *IbWRKYs*, and structural genes in various Chinese sweet potato cultivars

The expression levels of *IbMYB1*, *IbMYB27*, *IbMYBx-ZZ*, *IbbHLH2*, *IbbHLH1*, *IbWRKY44*, *IbWRKY21*, and *IbWRKY24* and structural genes *IbDFR* and *IbANS* were analyzed in Chinese sweet potato cultivars, including 6 purple- and 2 orange-fleshed cultivars, to investigate whether the involvement of *IbMYBs*, *IbbHLHs*, and *IbWRKYs* in the regulation of anthocyanin accumulation was common in purple-fleshed sweet potato (Figure 6A). The expression levels of *IbMYB1*, *IbMYB27*, *IbMYBx-ZZ*, *IbbHLH2*, *IbbHLH1*, *IbWRKY44*, *IbWRKY24*, *IbDFR*, and *IbANS* in the tuberous roots of purple-fleshed sweet potato cultivars were higher than those of orange-fleshed sweet potato cultivars (Figure 6B). The expression levels of these genes and anthocyanin contents were significantly positively correlated (Supplementary Figure 3). These results further indicated that anthocyanin biosynthesis in the storage roots of purple-fleshed sweet potato might be regulated by activators, including *IbMYB1*, *IbbHLH1*, *IbbHLH2*, *IbWRKY24*, and *IbWRKY44*, and repressors, including *IbMYB27* and *IbMYBx-ZZ*.

### Functional verification of IbWRKY, MYB repressors, and MBW/MBWW complexes

**FIGURE 7:**
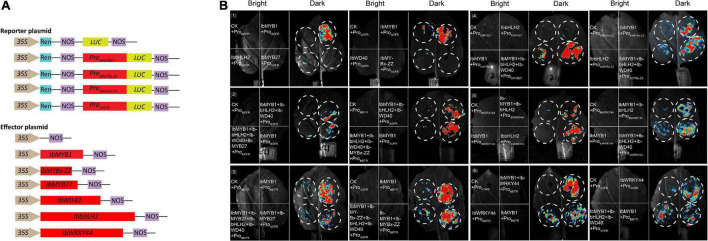
Identification of IbMYB1, IbMYB27, IbMYBx-ZZ, IbWRKY44, IbWD40, and IbbHLH2 transactivation activities of IbDFR, IbMYB27, IbMYBx-ZZ, and IbWRKY44 promoters by a dual-luciferase assay. **(A)** Schematic of reporter and effector constructs used in transient expression assay. **(B)** Transient expression assays showing that different TFs activates or represses the transcriptional function of promoters. Luminescence images of *Nicotiana benthamiana* leaves are shown 48 h after coinfiltration with the constructs indicated in the left. The red color represents strong signal, whereas the blue color represents weak signal.

Promoter activation tests were conducted by dual-luciferase assays to further confirm the activities of the screened TFs. Six effectors were generated by inserting 3 MYB (i.e., *IbMYB1*, *IbMYB27*, *IbMYBx-ZZ*), 1 bHLH (i.e., *IbbHLH2*), 1 WDR (i.e., *IbWD40*), and 1 WRKY (i.e., *IbWRKY44*) coding sequences into the pGreenII-62-SK vector. The promoters of *IbMYB27*, *IbMYBx-ZZ*, *IbDFR*, and *IbWRKY44* were cloned into the pGreenII-0800-LUC vector to generate a reporter construct (Figure 7A). Vectors were separately infiltrated into *N. benthamiana* leaves. As shown in Figures 7B[1,2], the *IbDFR* promoter was activated by IbMYB1 alone or in combination with IbbHLH2 and IbWD40. This activation was repressed by IbMYB27 or IbMYBx-ZZ (Figures 7B[2,3]). IbMYB27 had a strong suppressive effect upon *IbDFR* promoter activation when coinfiltrated with IbbHLH2 and IbWD40 but only had a modestly suppressive effect upon activation by IbMYB1 (Figure 7B[3]). IbMYBx-ZZ also had a strong suppressive effect upon *IbDFR* promoter activation when coinfiltrated with IbbHLH2 and IbWD40 but only had a weakly suppressive effect upon activation by IbMYB1 (Figure 7B[3]). Besides, IbMYB27 showed a stronger suppression affect than IbMYBx-ZZ especially when they were coinfiltrated with IbMYB1 alone (Figure 7B[3]). At the same time, the promoters of *IbMYBx-ZZ* and *IbMYB27* were activated by IbMYB1 alone or in combination with IbbHLH2 and IbWD40, and the activation effect of IbMYB1 together with IbbHLH2 and IbWD40 was stronger than that of IbMYB1 alone (Figure 7B[4]). In addition, the promoter of *IbWRKY44* was activated by IbbHLH2 alone or in combination with either IbMYB1 or IbMYB1 and IbWD40 (Figure 7B[5]). The promoter of *IbDFR* was also activated by IbWRKY44 alone, but the activation effect of IbWRKY44 in combination with IbMYB1 was stronger than that of IbWRKY44 or IbMYB1 alone (Figure 7B[6]).

**FIGURE 8:**
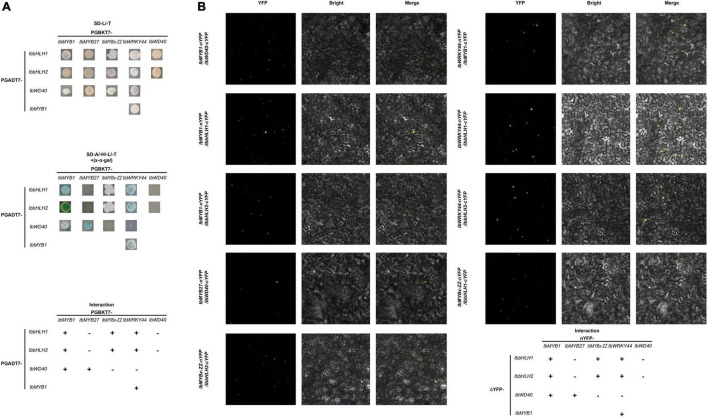
Interactions of IbMYB1, IbMYB27, IbMYBx-ZZ, and IbWRKY44 with IbWD40, IbbHLH1, or IbbHLH2 *in vivo*. **(A)** Interactions of IbMYB1, IbMYB27, IbMYBx-ZZ, and IbWRKY44 with IbWD40, IbbHLH1, or IbbHLH2 *in vivo* by a yeast two-hybrid (Y2H) assay. **(B)** Verification of the interaction of IbMYB1, IbMYB27, IbMYBx-ZZ, and IbWRKY44 with IbWD40, IbbHLH1, or IbbHLH2 *in vivo* by a bimolecular fluorescence complementation (BiFC) assay. Images show overlays of fluorescence and light views. “+” represents positive interaction between these two TFs, whereas “-” represents negative interaction.

Y2H and BiFC assays were performed to determine how TFs, including IbMYB1, IbMYB27, IbMYBx-ZZ, IbbHLH1, IbHLH2, and IbWRKY44, were involved in anthocyanin biosynthesis regulation. A summary of the interactions and their relative strengths is shown in Figure 8. The Y2H experiment result indicated that IbMYB1 interacted with IbbHLH1, IbbHLH2, or IbWD40 protein (Figure 8A). However, IbMYB27 interacted with IbWD40, and IbMYBx-ZZ interacted with IbbHLH1 or IbbHLH2 (Figure 8A). IbWRKY44 interacted with IbMYB1, IbbHLH1, or IbbHLH2. However, no interaction between IbWD40 and IbWRKY44, IbbHLH1, or IbbHLH2 was observed (Supplementary Figure 4A). These interaction results were confirmed by BiFC assays (Figures 8B; Supplementary Figures 4B,C).

## Discussion

The MBW activation complex, which is composed of MYB, bHLH, and WD40 (WDR) protein, is conserved in multiple plant species to activate anthocyanin biosynthesis (Gonzalez et al., 2010; Albert et al., 2014; Li et al., 2021b). The R2R3-MYB factors in the complex are the most crucial for the spatial and temporal localization of anthocyanins (Davies et al., 2012). *IbMYB1* controls anthocyanin biosynthesis in tuberous roots (Mano et al., 2007), and its expression is essential for the purple color of leaf and storage root in sweet potato (Zhang et al., 2021a). Based on the sequences of coding regions, *IbMYB1* amplified products from a purple-fleshed sweet potato, Ayamurasaki, were classified into two genotypes, *IbMYB1-1*-type and *IbMYB1-2*-type, whereas *IbMYB1* amplified product from its spontaneous mutant, AYM96, belonged to *IbMYB1-1*-type (Tanaka et al., 2012). Genome-wide analysis of expression quantitative trait loci (eQTLs) reveals one eQTL for IbMYB1-2 on chromosome 12 associated with flavonoid biosynthesis was the most promising candidate gene responsible for flesh color variation in sweet potato (Zhang et al., 2020). However, *IbMYB1-1* and *IbMYB1-2* shared the same coding sequences, only *IbMYB1-2* is identified to be responsible for anthocyanin accumulation in tuberous roots (Tanaka et al., 2012). Further cloning and sequencing of the flanking regions of IbMYB1 sequences showed that the large differences in the flanking sequences, especially the differences in 5′ flanking sequences resulted in the functional difference (Tanaka et al., 2012). To determine the genotype of IbMYB1, fragments of different sizes were amplified from the 5′ flanking sequence of *IbMYB1-1* and *IbMYB1-2* using specific primers (Tanaka et al., 2012). In our research, the same PCR assays were performed on ZZ*^P^* and its mutant, XL*^Y^*, the missing *IbMYB1-2* in the genome of XL*^Y^* mutant caused evident pigmentation and anthocyanin accumulation losses in its tuberous roots (Figure 1). This finding was similar to that observed in a previous report (Tanaka et al., 2012). Besides, the expression levels of *IbMYB1*, *IbbHLH2*, and structural genes in XL*^Y^* were significantly down-regulated compared with those in wild-type ZZ*^P^* (Figures 2B,C). Moreover, *IbMYB1* and *IbbHLH2* were highly expressed in tuberous roots of six Chinese purple-fleshed sweet potato cultivars, and the expression levels of *IbMYB1* (*R*^2^ = 0.56, *p* < 0.0001) and *IbbHLH2* (*R*^2^ = 0.7873, *p* < 0.0001) showed significantly positive correlations with anthocyanin contents of eight Chinese sweet potato cultivars (Supplementary Figure 3). Although the expression of *IbbHLH1* in XL*^Y^* was not significantly down-regulated compared with that in ZZ*^P^* by RNA-seq (data not shown), *IbbHLH1* showed high expression level in all Chinese purple-fleshed sweet potato cultivars (Figure 6). Previous research showed that PhJAF13 (bHLH1) and PhAN1 (bHLH2) are involved in the anthocyanin accumulation in leaf and flower in petunia and that the R2R3-MYB activator, PhWDR (WD40), and PhJAF13 proteins form an MBW complex to activate the expression of *PhAN1* (*bHLH2*). Then, the R2R3-MYB activator, PhWDR, and PhAN1 (bHLH2) protein form a core MBW complex to activate the expression of the anthocyanin biosynthesis genes and anthocyanin biosynthesis (Albert et al., 2014). *DcMYB7* controls purple pigmentation in carrot roots by regulating its *DcbHLH3* partner and the tested anthocyanin biosynthetic structural gene (Xu et al., 2019). Similarly, IbMYB1 interacted with IbbHLH1, IbbHLH2, or IbWD40 and might form a MYB1-bHLH1-WD40 or MYB1-bHLH2-WD40 complex in our results (Figure 8). Moreover, the MYB1-bHLH2-WD40 complex and IbMYB1 could activate the promoter of *IbDFR* (Figures 7B[1,2]). Besides the MBW complex, WRKY TFs are identified in some plants to regulate anthocyanin biosynthesis (Li et al., 2021a). For example, MdWRKY40, MdWRKY11, PyWRKY26, PbWRKY75, StWRKY13, and PhPH3 have been identified to promote anthocyanin accumulation (Verweij et al., 2016; Wang et al., 2018; An et al., 2019; Liu et al., 2019; Li et al., 2020; Zhai et al., 2021; Zhang et al., 2021b). In the present study, *IbWRKY24*, *IbWRKY44*, and *IbWRKY21* homologous to *MdWRKY11*, *PyWRKY26*, *StWRKY13*, and *PhPH3* (Figure 4) were found to be markedly downregulated in XL*^Y^* (Figure 2C). Moreover, *IbWRKY24* and *IbWRKY44* were highly expressed in tuberous roots of all selected Chinese purple-fleshed sweet potato cultivars (Figure 6). The expression levels of *IbWRKY24* (*R*^2^ = 0.7976, *p* < 0.0001) and *IbWRKY44* (*R*^2^ = 0.6272, *p* < 0.0001) were significantly correlated with anthocyanin contents in the tuberous roots of Chinese sweet potato cultivars (Supplementary Figure 3). Additionally, IbWRKY44 could activate the promoter of *IbDFR*, and the coinfiltration of IbMYB1 and IbWRKY44 showed stronger activation than IbWRKY44 alone (Figure 7B[6]). Previous research reported that WRKY TFs are regulated by the MBW activation complex in *Arabidopsis* and *Petunia* and that the R2R3-MYB activator, WDR, bHLH, and WRKY protein form a MBWW complex to activate anthocyanin biosynthesis (Gonzalez et al., 2016; Verweij et al., 2016). The MBW activation complex containing bHLH2 activates the promoters of *IbbHLH2* and *PhAN1* (bHLH2) in sweet potato and petunia, and this activation serves as reinforcement regulation (Albert et al., 2014; Deng et al., 2020). In our results, the *IbWRKY44* promoter was activated by MYB1-bHLH2-WD40 (Figure 7B[5]), and IbWRKY44 could interact with MYB1 and bHLH2, which were two members of the MYB1-bHLH2-WD40 activation complex. Thus, IbWRKY44 might form a MYB1-bHLH2-WD40-WRKY activation complex to activate anthocyanin biosynthesis in the tuberous root of purple-fleshed sweet potato. Moreover, the *IbWRKY44* promoter was activated by bHLH2 and not by IbMYB1 (Figure 7B[5]).

Unlike MYB activators that induce anthocyanin accumulation, a fairly large number of MYB repressors that inhibit anthocyanin accumulation have been identified in the last decade (Lafountain and Yuan, 2021). In petunia, as a member of Convolvulaceae, repressors *PhMYB27* (R2R3-MYB) and *PhMYBx* (R3-MYB) are identified to repress anthocyanin accumulation (Albert et al., 2014). Similarly, Deng et al. (2020) found that MYB repressor genes *IbMYB27* and *IbMYBx* are highly expressed in young purple leaves, but their repression functions are not identified in their research. In the present paper, *IbMYBx-ZZ*, which coded a novel R3-MYB repressor, and *IbMYB27* in the tuberous roots of ZZ*^P^* were up-regulated compared with those in XL*^Y^*, accompanied by the high expression of *IbMYB1* (Figure 2C). *IbMYB27* and *IbMYBx-ZZ* were highly expressed in the tuberous roots of all selected purple-fleshed sweet potato cultivars (Figure 6). The ectopic expression result showed that IbMYBx-ZZ and IbMYB27 repressed anthocyanin accumulation activated by IbMYB1 (Figure 5). Moreover, IbMYB27 and IbMYBx-ZZ repressed the activation of *IbDFR* promoter that was activated by IbMYB1 or MYB1-bHLH2-WD40 (Figures 7B[2,3]). At the same time, the promoters of *IbMYB27* and *IbMYBx-ZZ* were activated by IbMYB1 or MYB1-bHLH2-WD40 (Figure 7B[4]), and these activations allowed for the feedback repression of anthocyanin biosynthesis. In petunia, PhMYB27 interacts with bHLH and is incorporated or binds to MBW complexes, thus changing the complex activity from activation to repression (Albert et al., 2014). MYBx and other R3-MYB, as competitive inhibitors, bind the bHLH that requires the formation of MBW complexes (Kroon, 2004; Pesch and Hülskamp, 2004; Wester et al., 2009). IbMYBx-ZZ interacted with IbbHLH1 and IbbHLH2 in our result (Figure 8). In petunia, MYB27 shows repression affected on activation of the DFR promoter when MYB27 is coinfiltrated with bHLH and DPL(MYB), and repression by MYB27 requires the ERF-associated amphiphilic repression (EAR) motif and the formation of the MBW complex. However, in their study, the effect of MYB27 repression on the activation of the DFR promoter by DPL alone is not determined. In our results, IbMYB27’s suppressive effect on the *IbDFR* promoter activation by IbMYB1 or by MBW activation complex was tested. IbMYB27 had a strong suppression to *IbDFR* promoter activation by the MBW activation complex and only a modest suppression on the activation by IbMYB1 (Figure 7B[3]). IbMYBx-ZZ also had a strong suppression to *IbDFR* promoter activation by MBW activation complex and a weak suppression on the activation by IbMYB1 (Figure 7B[3]). These results suggested that IbMYB27, similar to PnMYB27, contained the EAR motif and mediated transcriptional repression by binding to the promoter of target genes either directly itself or as part of a DNA-binding complex (Ohta et al., 2001; Kagale et al., 2010), just the later pattern showed a strong suppressive effect. However, IbMYB27 might repress anthocyanin biosynthesis in the tuberous root of purple-fleshed sweet potato by inhibiting the formation of active MBW complex *via* binding to WD40 (Figure 8). Thus, MYB27 had a potential double-lockdown mechanism for reducing anthocyanin production. However, IbMYBx-ZZ, a R3-MYB factor containing a single MYB repeat and lacking any repressive motif, asserted its repressive function only through competition for WD40 partner with IbMYB1 (Figure 7B[3]).

**FIGURE 9:**
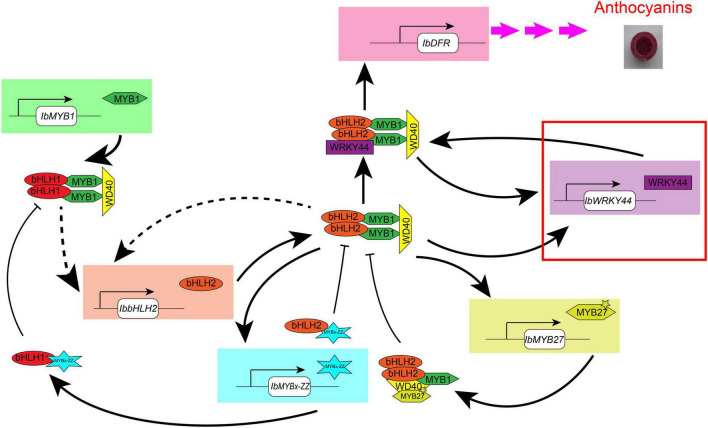
Model of the anthocyanin regulation network in tuberous roots of sweet potato. This model is modified in accordance with the method of Albert et al. (2014). *IbMYB1* is expressed and then forms a MBW activation complex with bHLH1 and WD40, which activate the expression of *IbbHLH2*. Then, a core MBW activation complex containing bHLH2 forms combines with WRKY44 protein, forming a MBWW complex, and activates the expression of the anthocyanin biosynthesis genes, ultimately resulting in anthocyanin accumulation. The MBWW complex can reinforce the expression of *IbWRKY44*. The core MBW activation complex can also reinforce the expression of *IbbHLH2* and activate the expression of *IbWRKY44* and MYB repressors, like *IbMYBx-ZZ* and *IbMYB27*. Then, MYB repressors collaborate with bHLH and WD40 protein and inhibit the expression of anthocyanin biosynthesis genes. Arrow with solid line represents regulation proven in sweet potato leaves, and dotted arrow represents the regulation that has not been proven yet. The red rectangle represents novel TF that we found to be involved in anthocyanin biosynthesis in sweet potato.

Overall, through phenotype, genotype, and transcriptome analyses carried on the purple-fleshed sweet potato ZZ*^P^* and its mutant XL*^Y^*, the candidate TFs that involved anthocyanin accumulation in the tuberous root of purple-fleshed cultivars, including IbMYB1, IbbHLH2, IbWRKY24, IbWRKY44, IbWRKY21, IbMYB27, and IbMYBx-ZZ, were screened. Given that TFs shared a common expression pattern in various purple-fleshed sweet potato varieties, the anthocyanin biosynthesis of tuberous roots was likely regulated by IbMYB1, IbWD40, IbHLH1, IbbHLH2, IbWRKYs (i.e., IbWRKY24, IbWRKY44, IbWRKY21), IbMYB27, and IbMYBx-ZZ in sweet potato. Further functional verification of the above TFs was conducted by Y2H, BiFC, and dual-luciferase assays. These tests showed that IbMYB1 was the central determinant of the MBW complex and that IbbHLH2, IbWRKYs, and repressors IbMYB27 and IbMYBx-ZZ were activated by the MBW/MBWW activation complex (Figure 9). This regulatory network provides reinforcement and feedback regulation to maintain the level of anthocyanin accumulation in the tuberous roots of purple-fleshed sweet potato similar to other plants (Albert et al., 2014; Verweij et al., 2016; Peng et al., 2020).

## Conclusion

The comparative transcriptome analysis of purple sweet potato and its yellow mutant were carried out. The missing *IbMYB1-2* caused a dramatic downregulation of the expression of TFs *IbMYB1*, *IbbHLH2*, *IbWRKY24*, *IbWRKY44*, *IbWRKY21*, *IbMYB27*, and *IbMYBx-ZZ* and structural genes and ultimately resulted in a dramatic decrease in anthocyanin content. The functions of repressing anthocyanin accumulation of *IbMYB27* and *IbMYBx-ZZ* were identified by transient transformation in tobacco leaves. The expression patterns of TFs and structural genes in the tuberous root of Chinese sweet potato cultivars showed that the expression levels of *IbMYB1*, *IbbHLH1*, *IbbHLH2*, *IbWRKY24*, *IbWRKY44*, *IbMYB27*, and *IbMYBx-ZZ* were significantly positively correlated with anthocyanin contents in tuberous roots. Further functional verification of the above TFs was conducted by Y2H, BiFC, and dual-luciferase assays. These tests showed that the MBW/MBWW complex activated the protomers of anthocyanin structural gene *IbDFR* and the promoters for *IbWRKY44*, *IbMYB27*, and *IbMYBx-ZZ*. These results indicated reinforcement and feedback regulation and provided valuable information for the regulation of anthocyanin biosynthesis in purple-fleshed sweet potato.

## Data availability statement

The original contributions presented in the study are publicly available. This data can be found here: NCBI, PRJNA837528.

## Author contributions

WD, LT, and XH designed the study. WD, LT, and YP performed the experiments. YL and YQ analyzed the data. WD wrote the manuscript. XX and XH funded the research project. All authors read and approved the manuscript.

## Footnotes

1
https://www.ibm.com/analytics/spss-statisticssoftware

## Supplementary material

Supplementary Figure 1 Volcano plots and KEGG classification of DEGs from ZZ*^P^* and XL*^Y^*. **(A)** Volcano plots displaying the upregulated (red), downregulated (green), and unregulated (blue) genes in XL*^Y^* compared with those in ZZ*^P^*. **(B)** KEGG enrichment of DEGs between ZZ*^P^* and XL*^Y^*. The X-axis represents the rich factor corresponding to the pathway, and the Y-axis represents the pathway name. The size of the *q*-value is represented by the dot color. A small *q*-value indicates close color to red. The number of DEGs contained in each pathway is represented by the dot size.

Supplementary Figure 2 Phylogenetic analysis and protein sequence alignment for putative MYBs with function-verified homologous genes of other plant species. **(A)** Phylogenetic relationship of putative MYBs in sweet potato and function-verified anthocyanin-related MYBs in other species. Orange- and green-shaded areas indicate R2R3-MYB activators and repressors, respectively. The blue-shaded area shows R3-MYB repressors. The pentagram represents *IbMYBx-ZZ*. **(B)** Protein sequence alignment for the putative MYBs with function-verified homologous genes of other plant species. Black lines represent the R2 and R3 domains.

Supplementary Figure 3 Correlation between contents of anthocyanins and relative expression level of nine DEGs in the tuberous roots of sweet potato. The X-axis represents the contents of anthocyanins, and the Y-axis represents the relative expression level of the gene.

Supplementary Figure 4 Self-activated test of IbMYB1, IbMYB27, IbMYBx-ZZ, IbWRKY44, IbWD40, IbbHLH2, and IbbHLH1 in **(A)** Y2H and **(B)** BiFC experiments. **(C)** No fluorescence signal detected in IbMYBx-ZZ-IbWD40, IbbHLH1-IbWD40, IbbHLH2-IbWD40, IbWRKY44-IbWD40, IbMYB27-IbbHLH1, and IbMYB27-IbbHLH2 pairs.

Supplementary Table 1 Primers used in this manuscript.

Supplementary Table 2 Summary of RNA-seq data.

Supplementary Table 3 Promoter sequences of *IbMYB27*, *IbMYBx-ZZ*, *IbWRKY44*, and *IbDFR*.

Supplementary Table 4 Sequences of *IbMYBx-ZZ*, *IbWRKY21*, *IbWRKY24*, and *IbWRKY44*.

Supplementary Table 5 Protein sequences used in Figure 4 and Supplementary Figure 2.
