# Supplementary figures and images for: Comparative transcriptome analysis of purple-fleshed sweet potato and its yellow-fleshed mutant provides insight into the transcription factors involved in anthocyanin biosynthesis in tuberous root
Source: Front Plant Sci. 2022 Aug 8;13:924379. doi: 10.3389/fpls.2022.924379 (PMC9393619; doi:10.3389/fpls.2022.924379)

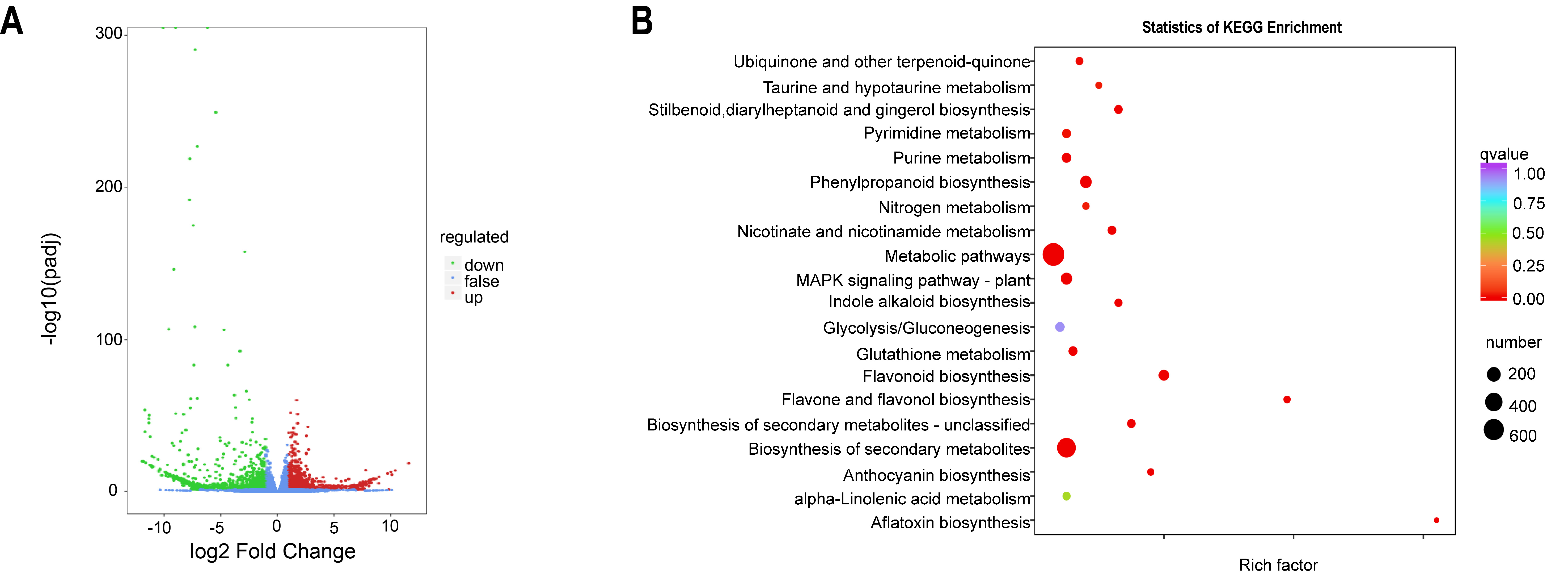

Supplement: Supplementary Figure 1 — Volcano plots and KEGG classification of DEGs from ZZP and XLY. (A) Volcano plots displaying the upregulated (red), downregulated (green), and unregulated (blue) genes in XLY compared with those in ZZP. (B) KEGG enrichment of DEGs between ZZP and XLY. The X-axis represents the rich factor corresponding to the pathway, and the Y-axis represents the pathway name. The size of the q-value is represented by the dot color. A small q-value indicates close color to red. The number of DEGs contained in each pathway is represented by the dot size. [file Image_1.JPEG]

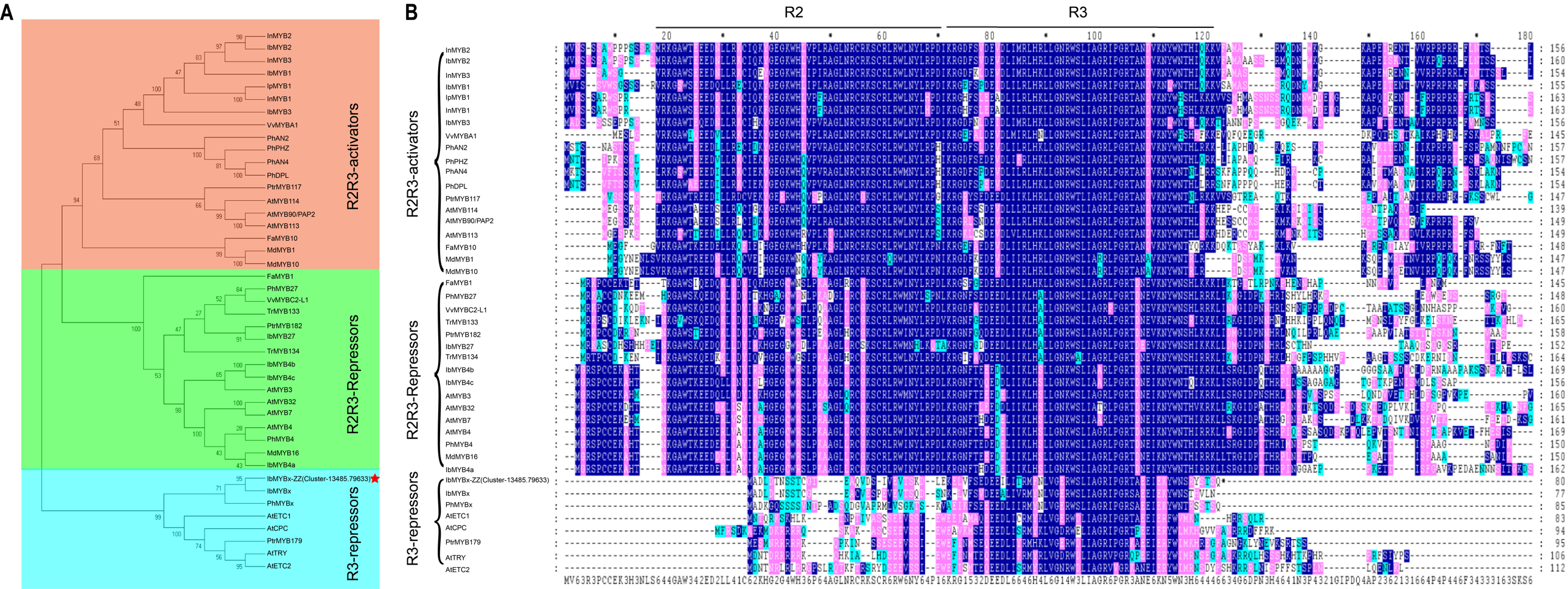

Supplement: Supplementary Figure 2 — Phylogenetic analysis and protein sequence alignment for putative MYBs with function-verified homologous genes of other plant species. (A) Phylogenetic relationship of putative MYBs in sweet potato and function-verified anthocyanin-related MYBs in other species. Orange- and green-shaded areas indicate R2R3-MYB activators and repressors, respectively. The blue-shaded area shows R3-MYB repressors. The pentagram represents IbMYBx-ZZ. (B) Protein sequence alignment for the putative MYBs with function-verified homologous genes of other plant species. Black lines represent the R2 and R3 domains. [file Image_2.JPEG]

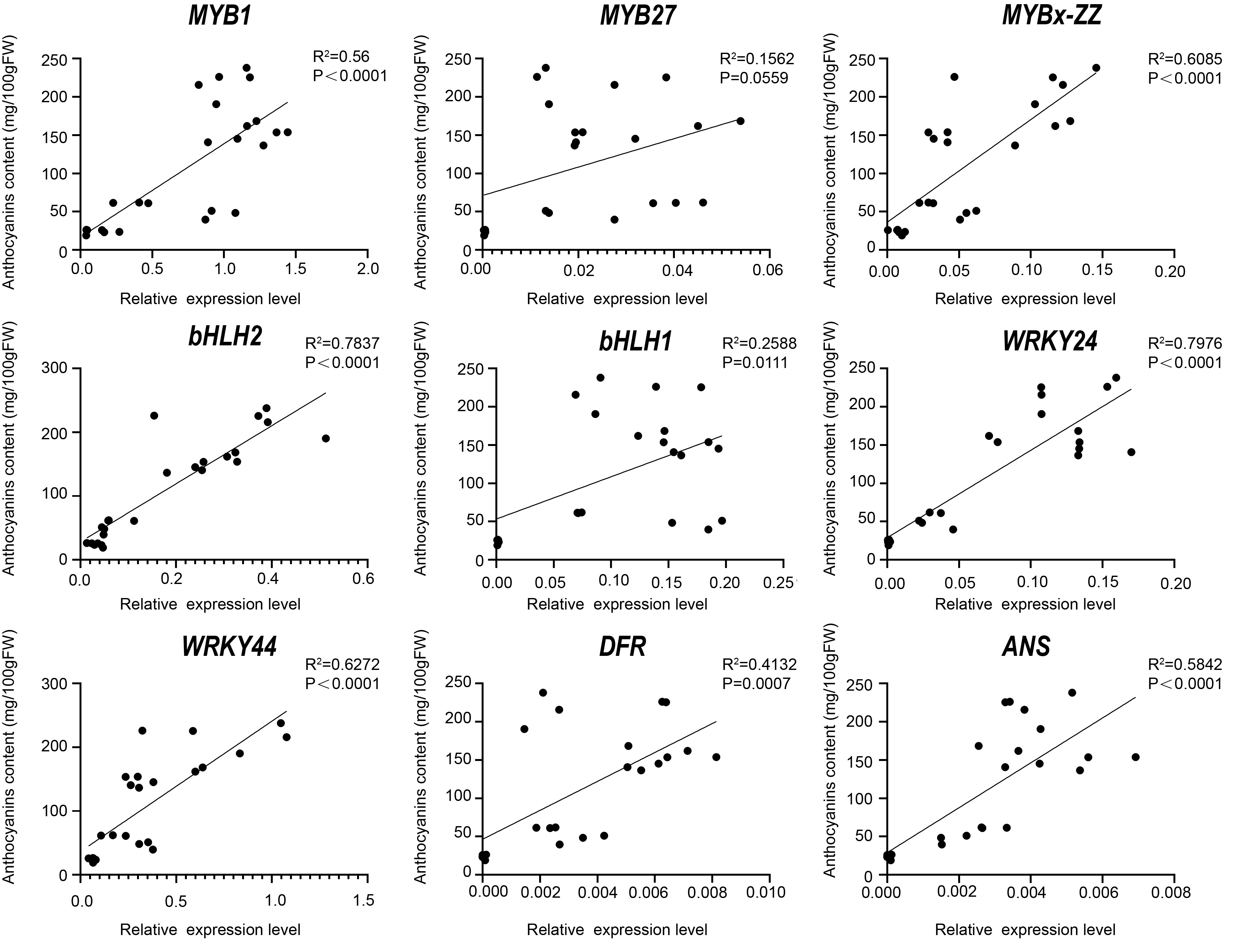

Supplement: Supplementary Figure 3 — Correlation between contents of anthocyanins and relative expression level of nine DEGs in the tuberous roots of sweet potato. The X-axis represents the contents of anthocyanins, and the Y-axis represents the relative expression level of the gene. [file Image_3.JPEG]

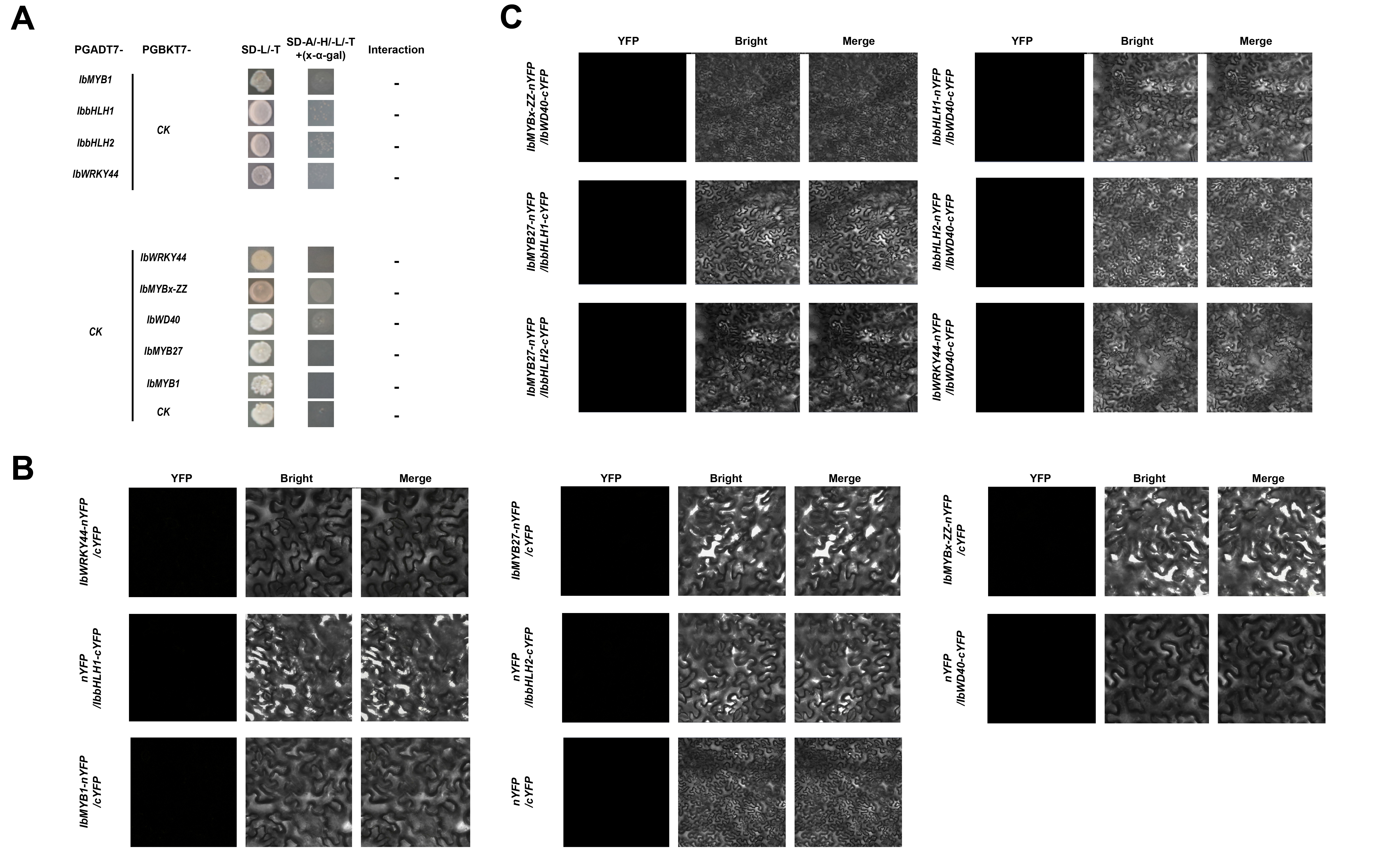

Supplement: Supplementary Figure 4 — Self-activated test of IbMYB1, IbMYB27, IbMYBx-ZZ, IbWRKY44, IbWD40, IbbHLH2, and IbbHLH1 in (A) Y2H and (B) BiFC experiments. (C) No fluorescence signal detected in IbMYBx-ZZ-IbWD40, IbbHLH1-IbWD40, IbbHLH2-IbWD40, IbWRKY44-IbWD40, IbMYB27-IbbHLH1, and IbMYB27-IbbHLH2 pairs. [file Image_4.JPEG]
