# Supplementary material for: Comparative transcriptome analysis of purple-fleshed sweet potato and its yellow-fleshed mutant provides insight into the transcription factors involved in anthocyanin biosynthesis in tuberous root
Source: Front Plant Sci. 2022 Aug 8;13:924379. doi: 10.3389/fpls.2022.924379 (PMC9393619; doi:10.3389/fpls.2022.924379)
Supplement: Supplementary Table 3 — Promoter sequences of IbMYB27, IbMYBx-ZZ, IbWRKY44, and IbDFR. [file Table_3.DOCX]

1.Promoter sequence of *IbMYB27*

TGGTGGAAAAGGACGGCAGTGTCTGAGGCTCGCTGCAGACCTGAAGCTTGATATCGAATTCGCGTGTCGCCCTTGTGGATTGTCACTTATAAATCTATGACAAAAAATGGAATTAACTTATATATAAAATATAAATAGTTAATTCAGAGTTTTGGTCCTAGATTTATAGGTATCATTTTACTTGTAGTCTTTTTTTTAGAACTTCCTTGTTGGTTTTAGTATTATTCTGGCATAATAATTTTAGTCATCTGTCAATAAATATGTTTAAATGGCAATAAACATGAGGGACATTTTGGTCTATTTAGCCTACAATCTTCTCAACTCCACTTTGTTATAAGAGTTAATTCCTGTTTTAGTCCTAGATTTATAGGTGTCATTTCACTTTTGGTTATAGCATTATTGTGGCATGATCATTTTTTGTCCTTCATCAACGAATTTGTTTACATGGAGTTAAAAACGCTAAAAACAAAAGTGGAATGGCACCTATAAATCTATTACAAAAAAAATAAAATAAATTAACTCTTAGAACAAAGAGAAGTTGAGAAGATGGTAGGCCTAATAGACAGGGATGTCCTCTTTTAAACATATTTTTTGACGGAGAACAAAAAAAGGTCATACTACAATAATACTAGGTCCAACAAAGGATGTTCTGAAAAATTAAAAAAAAGGACTAAAAGTGGGATGACATCTATAAATCTATAACAAAGAAAAAAAGAAAAAAGAATTAACTCAAATATAAATGTGTAGTCCATTTATCCTTTTTAACTTTTAATTGAACCGAAAAACATTATTCAACTTTTTTCTATTAATTAAGCATATAATATGTAAATAAAATATAAATGTGTAACTTAATTATATATTTAAATTTTTTTCTTTCAAAATAAATATATATTTGAAATTTTAATATAGAAGGAATATAACATTCAATTGAGCACATATTATAGTGCCCGTTATTAGTTGTAGTGTTGTGATAAATACACATCACAAATATAATAAACATCGGTCCCAATCCAAAGCTCATTGTATTATATACATAGCGCCTCGCTGTCCCAAGTTTATCAGGACGTAAGCTACACTCAGTTGATCATTTATACTTTATAACAAAACTAAATGGTGTTTATTTGTGGCTTAATAGAGTCGATAATACTAAAAAGAAAAAAAAAGGAGGTTTATTTGTCATAAAATTTGCACAGACTTGATATTAATTTTATATTTTCATCCAAAAAGATAATACTATTATGGGTCACACTTGTGTGAGACCGTCTCACGAATCAGATGGGTCAAGTCAATATGCAATTATAATACTTATATAGCAAATGTAATACTAATTAAGAATAAAGTGTTTGTTACTTATAATGGAAAATGTAATATTTTGAAGGCAAATGTAATACTTATATAGCAAAATACGTACGTACCTATGTAATTGTCATCTAGCAAGTGTTGATAATACTTGTCCATGCAGAAAATGTAAAATTTTCTCGTCCTCACATCATGGTCCAACAAGATAGTAGAAAGGTAAACCAGGGTGACTCAATTAATCAACTGACGTTAGACTTTTACTCACTATGCACAAAAAAAACCCTCTCATTTTGAGAGGTTATTCTCACACGCTGGTGTCTATTGTGACAAATTTCATCTGTGTGATCAATTGAGTTACGCATGTGTACTGGGTGCAACTCTTACACTACGATTGCTAGAGTTTGATTCTGTATCATTACCTATAATTAACCTACTTACGAATCAACTGAGTTGCCTGTGCGATAATAATTGCTCGTGCATTATAGTTGAGGTAGTACCATAGAAAGAAAAATTGTATTGATTATCAATTTTATAAAAGTGGGTGGTGTGAGTAAGGCATGCATTGCATACATACTATAGCATGCAGGGCCAAACCACCGGTCAACTCTCTCATGGTCGTCCTCCTTTTATTCCATAAAACACTATAAAGTGTAAACCACCCCCACACCACACCTCACCCACCGCGCCTCCGTCTTCAAGCAACTATATATAATTACACTCTCATCATTTTCTATACATACATACGACATATATATAGCCAAGGAGTGTTTTAATTTCTTCCCGGATCCACACACCTAATTCACTTCTTGTCTCTCCGACAACTAACGACTATGAGGAAGGCTTCCTGCGACCATTCTCATCATCATCATCATGAGATCAACAAGGGTGCTTGGTCCAAGCAGGAAGACCAGAAGCTCCTAGATTACATCCGAAAACACGGCAAGGGCGACACGCGAATTCGATATCAAGCTTCAGGTCTGCAGTCAATACGACGACTTTCCCTATG

2.Promoter sequence of *IbMYBx-ZZ*

CGTATCATATTCCTGAATATGCAATTTTTATCATATTCCTGAATATGCAATTTTCGTGTTAATATTAGTCTGTGTAAAGATTAATTGATTGTTGTAATATCGTAGGCCAACGATTTTACACCGGATATTGATGTATCACAACTATAACTTGTACTGGAAATTAAATGTGAAATGGATCCAATTAACATGCTTTGGACCCGCCTATTCCTTTAATTTATGGAAAAGTCAGCAGTCATTAATTTATTGTGTTATATGAATGAAATTTACCTTATGACTCTAGGTGGCAAAGGATTAAATTATTAACCTATATATCAAAATCAAGAATATCAATAGACATCTACGATGAGTTAAATGTAAAACTTTATAATTATTAAATTAATTATTCCAGGAAAAATAGTCAAATAAGCCCATGAAAGGAGACCAGTGGTATGGTGGTTGCCTTCCGCCAGCGTATGGCGACCGGTGACGGTTCTGCCATCGCCGGACGTCGATTTTTAGATTTCTTTATTTCAATTTAACATATTAAAAGGTCTTATTGTACTTTTTAAATATTTAAGGACTTACTTGACTTCTCAGATAATATTTAAGGACATATTTGACTATTTTCTCATTGTTCAATTAACTTGGTTGACAGCTGCATGGATTGACATGTTCTAATCCATGCAACTAATAGTCAATAAATGGTTGCTGTCATGTGGCAACTAAATTCCACTGCACCTCACTCAGCCGGAAGACTGTTTTGTATTTGTATTATTGTGAATTTGACAGCGATTTTATCCCCTGGCTGACTAGACTTCACTCAGTTCACTGTATTCCTACCAAGCCTTATCTAGAGTGTGACACAGTGACAACGATGTTTCTTTGTTTTCACACTCATAATCCTAACAACATAATTATACTTTTTTTGTATTACATTAAATATACTAAATAAACACAAACAAATATATGTAATCTAACATCATTAACTAATTACACAACTAAAAATTACATGACAACTAACATGTTCGTGTCGGGTAGGTGAAAAAACAAAGTAGATGAAAACACGCTCTCTAAATCTCTAATAATTTTTAAATTAACTACCTTCTTAAAGTTTAAACCCAAAATTTTTACCAAAAACTGCATTATTTATTTCATTCCCACATATTTTTTATTAGTTGGCATAGGATAGACACCTTATCTTTTGCTTTTAATGCTTTTCGGATATTTCACAATTAGCATATGGTGGGCACCATGCACCCCCCCTGTTGTTGCATCAAAATTTATTTGAGAAAAGTACACAGCCCAACAGTTTTGGTTTACCAGGTCCACGTGTCTAATGTCTAACATTTTCTTTTTATTTTTATTATTATTCATCCGTTATTCATGAATTAAATAACGCTATTATTATAACATATCGCATTTAAAATTTTGTATTAAGACGCTAATCTTGTAAAAATGAAAGTGACAACTGGTACTATATACGCTGGAAAACGACATGAGAAATTAAGGATAGAAAGCAAGTCGTATTCGGATAGAGTAGTACACTAAACAACTACACAAGGCTCTGTTCATTGGTTGCCAATACAGTAAGTACTAAACACTAAAGAATACAGATAGGATAGGACATGGATGGATATGTACTTGTTGGACATATATGAAGCAACTACATTTTAGGCTCATGAATACACTAATCATGTTTCTGCAAATTGATCATCCTAAATCCCAATTAAGTAGTGATTATGTTTGCATAATTGCTATATTAATGAATAGAACAAACAATCCCAGTGTGATGAAGGAGACAGATGAATTTCATTCTATGTTTTCACCTACAGAAATGGTAATAGATCTCAATGATTAATGTGATTGTCTGTAGAATATTATCCATTTTTTTAAATTTAAAATTTAAAATTTAAAATAAAATTAAAAAAAAGTCCCATGACCAACCACAGGAGGCATATGCACCCAACTACCAACCTATCAAGAAAGTTGGACCACCACAACTACCATTCATGAGGTTTTGGCCTTTTATCAACTCCCATTCTCCTGCAAATTAAAATTCCCAACTACAAAGCATAAAATTCAAGACTTTTCTTGGGATTGATTCTTTTTTTTTCTCTTTCTTTATTATTATTATTTTTTTTATTTTTTTTTCTCTCTATTTATATTGTAGATGTTGTTGGCTTATCCATAGCAGTTGCCTCAATACTTCACCTTTTTGGCTACTATTCTGCTGCTTGTGTTTTTGACAATATTTTCAACTCCCATGGCTGACTTGGATACCAATTCAAGTACTTGTGGTACGATACCAATTCAAGTACTTGTGGTAATG

3.Promoter sequence of *IbWRKY44*

GCCAGGTTGTCATCATTATTGCGGGAAATTATATATGATCAAATTACAAGTTATGAATTAAACATGACCAAACTTTATATAACCATGGAAAAAATGTTAGAAACTAATGTAAAAGATCCATTCAGGAGTTTATATGTCTCGGGTTCAAGCCTTAAAAATACAGTTTTGAACATAATTAGTCTTAATGTATAGAATCTAGTATTTCAATAAAAATAATGATGACTTTAAAAGAAATGTTCGAGTGTATGAAATATAATTCTCGTATTAAAAAATTACGAATTTAGTCCTTACTAGCTATTTACACTTTCTTGATCCTAATGTGGAACTGCGGTTTCCATAGTCAATAATAATAATAATAATAATAATAATAATAATAATAATAATAATAATGACTAACTTTATCCAATAGCCAAAAATAGAGTGAGTTGGCCACTCATTTGGGAGGTTCTTTTGGCTCAAAGCTAAAATCCAAAAAATAAAAGTAAAAACAAGCCTATATAGAAAAACACCCATACTTATTGGGTAGATGTTGTTTAGAGAATTTTTTTATGTGACATAATATCATTTTTATAACTTATAATGTATATTATTCTACAACATAATACATATTATTATAACTCATAAAATATATTATTTTACCATAAAAATTTCATTATTCTAATATACAAAGTACATTATTTTAACTCATAAATTTCAACAACAGTCTTTTTGGACCGTGGTCGAAGTAGCTGTATTTGTCGAAAAACACTAACAAAATCCCATAACCCATAGGTCATTGTTCCAAGTCGCAATCAAAATGCCATAAATCTTTAGCACAAAAATGAAGGAAACATCAGTGTCATGATAAGGGCAACCATTTCTGCACAGTGCAAATCAACAACTATCAGTCTTCTACTCTTCTCTTTCTCTCTCTGGGTTTGGTCATTGGTATCTTGGTGAGCATTTCAAGATTCTGTCTGGGCTTTATTTGTTCTTTTAGTGTCGTATTTTTAAAATTGAAAATATTTTCCAGATATTAGTGTCTCTCTCCCTTTTTAAATTATTTTTCCTGTTGTTGGATTCTGGGTTTGTAAGTCTAATCTTTCTTGTCTGTAGGTAAAATTGAATGCTTCTACAGCTCTGTTAAGTGCACAGGCCTCATTGTAAGTCTAATCTTTCTTGTCTTTCTGTTTTTCATTCCAATTTCCAATCTTAATTTTCCCAAAGAACATATGCATGCTATTTAGGTTCACTCTTTGTGGTTAAAGAAAAGGTCTTAATTTCTTTAAAAGATTAGTTTGTGTGAATTGTTGTTGCTTTGATTTGCTTTTAGATTTTGCATTTGGATAAAATCTGGGGTAGTCTGATCTTTCTGTTTATCATTCCAATGTCAATTTTCCAAAAATATATATACATGATATTCAGGTTAACTTTGGGTCAATTGTTGCTTTGATCTGCTTTAATTTATAAGATTTTGCTTCTGTTTTTCATGTTAATATGGAGAATTCGGCTTGGGATGTTCTGAAAATACTTCCTAGATGGCTCTGTTTGATAATTCCCTCCTTTTTTTTTGAAGAAGAAGAAAACTATAATTCCCATTAGTTTCATCATAAGAATTATAGATCTTGCCAAAGACCTTGTAGTTGTAGTCTTAGGTTCAATTGATTCCCCCTTAGATGTAGCCAAAAATTTGAGAGAGAGAGAGAGAGAGGAGGAGGAGGATGAGGAGAAAGAATGATGGTTCTTGATTTATTGATTGCGGCATAAAACATGTGTAATATTTGAGGTTATATACGTATATTCTGTTAAGAATCCCATATTGAAAAATAAGAGAGAAGCATATGAGTTTATAAGGATATGAGTTAGACTAACTAATTGATTAGATTCAGTCTTTCAGGGTGGTTTGATTATAATATAATCAAACTTGAGATATCATGTGATGCATTAGTAGTTTGGTGTTGTTATCCCAATTAGACGATTAGTTCAATCTCATGTATGAGTTGACAGTAACCAAATTTTGTTGAAGATGGAGATCAAAGAGGTCAAGAAGATTGCGGTAGCCAAACCTGTGCACGTGTACATAATG

4.Promoter sequence of *IbDFR*

ATTAAGCGTTGAAGTGTGGTTATAAGTACATAAATTCATTAGTGTAGATATATAAATTACTATTGTATGTACATAAATTCACTACTGTAATTATTTAATTTACATTCACTATTGTTGATTTGTTGGTGTCATTTTATGTATCTACCGTATTATTTTGTTGTACACCTTCTATATCTAAAAGGAGAAAATAATGACAAATTCTCTCTCGATAAAAATGGGAAAATAACTCAATATAAACTCTCTAAGTATAGGAGGAAAGATTAGAATGATTTGGGATGATAAGAATGACAAATGACTACTCTATTTATAGTTGAAGCTTAGGGGTCAATTTTATAGATAGCCTAAAACTTAAGGACCAAATTAAATAAATGACTACCCTATTTGTAACAAATAATTTATAATTGCTTATTAAGTCACTGGCATCAGCCATTATATTAATACTGAATGGCAAACATTCCTCAATCAAAAGTCAGTATTCAACATGTCAATCAAATGCCAACTCGATCGTATATATGATTAAAGAAAATATCCTTTATGTAATTTGAATATTTGCTTGAGATTAACTTATAACAACAAATCATCAATAAAAGGAAAATTTGTACTTTTTGTCCATAAATTATAAGATAATTGTAGAATTCATTCCTAATTATTGATCGTGTTCATTTTTTCTTCTAATTTATCATTGGTGTTGCACTTTTCATCATTTTATTAACTTTTTGTGCTCACTTTTCGTCCCTAAGCTATACTAATGTTGCAAATTTCGTCCCTAATATTTTGTTAATAAAAAAACAAAAAATGCATCGCCAATGGTAGCTTAGAGACGAAAAGTGAGTACAACCAATTTAGGAGCGAATTCTAACAATTATCTTTAACTTAGGGACGAAAAGTGTAATTTTTCTGTCAATAAATGATAATAAGGGAAAATGTCACTTTGGGCATGCTTATAGCACTTTTTTCCCTGAATTATTCATATATCTATATTATTATTTTGAAAGCAGTGCTTTTCCCTCTTTTATGTTAAATGGACGTTTGGATTGGTTAAAAAAAAAGGGGCAAAAATAAGTAATTTCAAGGTTTTTTTCTTTCTTCAAAAAAGTTATTGTTTAATAGGTAAAATTCAGAAATAAAAGGGAAAAAAAAAGGAGCAACCAAATTAAAGCACACCTAAATTATGTTAGTAGAATTAGCTTAAAAATTATAATTATAATATCTACAATGGATGTCTATTTTTTTGTTGTATTTTTTTTAATCTCACATATAAGCAATAATTTGTTTGAGAAGAAAAACGACTTGAAATACCTATTTTGTCCTTATTTGTACAAGTGCTCATTTTACAATAGCGGATCGTCACTCTTTGAAAATAACGTAACTAAGGGATAATGTAAGTACAGGGCATGAATGATTCAGGAGGAAAAGTGCTATAAGTATAAAATCATGCATGAAGAACAATTGTCATTAAAAAAAAAAAGTGTCAAGTTGACCCTGGCCCTTATGTGTAATGAATAGTGAAATTAAGTCTTGTAGCTCAAAAAACTCCAATGAATTATTCTAAAGCAATAAAATAGTGCAAGTAGTTCATATTATTACCCATTAAGCGTTGAAGTGTGGTTAACCATGCTAACATGTGGTGTTTTAATAATACGGAGTAATAATTATTATTATTATTGTTCCAATAATAGAATTAAAGGTTGTGTTGTTGGGTCACACCAAACGGGGGGTTGATGTCGAGTATGAAGCATGCACGCACGTGCTTGGGTTTTGAGCCTATCTATATCGACGAGCGGACCAAACTGTCATGGCCAACTGAACGTGTATAAAACTTTGATCATTCTATAAGGCTTTTCACTTGCAACAATATATCGACAATTCAACAACCTTAATTATACTTTAATTTCTTCGCTTATTGATCTGAGCAAAATG
