# Supplementary material for: Comparative transcriptome analysis of purple-fleshed sweet potato and its yellow-fleshed mutant provides insight into the transcription factors involved in anthocyanin biosynthesis in tuberous root
Source: Front Plant Sci. 2022 Aug 8;13:924379. doi: 10.3389/fpls.2022.924379 (PMC9393619; doi:10.3389/fpls.2022.924379)
Supplement: Supplementary Table 4 — Sequences of IbMYBx-ZZ, IbWRKY21, IbWRKY24, and IbWRKY44. [file Table_4.DOCX]

1. > *IbMYBx-ZZ*

ATGGCTGACTTGGATACCAATTCAAGTACTTGTGGTACTGAAGCTCAAGTGGATTCTATAGTTGAGGTCACAAGTAAAGATTTAGAAAAGGAGCTTGTGTTCTCAGAGGATGAAGAAATCCTTATTACCAGAATGTTCAACTTGGTTGGAGAAAGGTGGTCGCTGATTGCTGGAAGAATCCCAGGAAGAACTGCAGAGGAAATCGAGAAGTACTGGAATTCAAGATACTCCACCAGCCAATGA

2. > *IbWRKY44*

ATGGAGATCAAAGAGGTAAAGAAGATTGCGGTAGCCAAACCTGTTGCGACGAGGCCTACTTGTTCTGCCTATACACCGTTCTCCGAGCTTCTTTCTGGTGCAATAAATGGATCATCCACCGGGGGCTGTTCTCAGACCGCAGTCATAGCTGCCATTAGACCGAAGACAGTGAGATTAAGGTCAGCGGGGAACCAAACTTTAGGTGGAAAGGTCGAGATGCCTGGCGTTTCAGCTTGGTCTCCTCCCGCTAATGTGTTGAAATCCGATGACAAACCAACCATCGTATACAAACCCATGCCAAAGCTACTCTCCAAGGCAACTTTTCCTCAAAATTTAAACATGAGAAGTTCTACATCGAGTCAGCAAAACGAAGCAGCTGAGGAGACGAACCAAGTCAGACCATCTGAGATCAGATTAGAAGCTCATCAAAGTTTATCATTGAAGTCAGGAACAGAGAAAAAACCGGTTGAAAACTCGAAAATGGCATTGCAGAACATAGAAGAGGATGAGCGTTCTGTGTTTCAGGGCAGTGGCGTGGACTGTCTTTCGAGCGATGGATATAATTGGAGGAAATATGGACAGAAGCAAGTTAAGGGAAGCGAGTTCCCGAGAAGTTACTACAAGTGTACACATCCGAAATGCCCCGTGAAAAAGAAGGTCGAGAGATCATCACTAGATGATCAGATTGCAGAAATTGTGTACAAGGGCGAGCATAACCACCCTAAGCCTCAGCTTCCAAGAAGCAACTTGCGGGATGGACAACCGAAAGGCGTTCTCGTCTCTGAGGATACTTGTAATGAAACCAATAACCCCGTGAGGAGTGAGCAACTTACTCTACAAAATGAACCTTGTGGACTTAGCACCGAGCATAAAAACACCACCATGTTGTCCACACGCTCAACTTATTCTAGCGGACCTCCTCCACCGTGCTATCCCGTTACATCTGCTGCAGCATTCCATGGCGCTGTATCAACTCCCGAAAACTCTTGCACTCCAAGTGGAATACATCGAGAAGGATTGGAGGCAGAAGGCGATGAGCTAAAAGGAAAAAGAAGGAAGTGCGGGAGCCAAACCAACAATGGAGCGACACTCGGGAATGGTGCAATGGAGACACAAACTGTTGTCGGGAGTACAACTGATTCTGAAACCACGGGGGATGGATTCCGCTGGCGAAAGTATGGGCAGAAGGTAGTTAAAGGAAATACGTACCCCAGGAGCTATTACAGATGCACGAGTCCTAAGTGCAATGTGAGGAAGTATGTGGAGAGAGCCCCCGATGATCCAAAATCCTTCATAACAACCTACGAGGGAAAACACAACCACGACATTCCAACAAGAAACCCAAATCCCGAAGCATCGAGATCAAGCACAAGGGCTGCAGCCACAAAGGAAAAATCATAG

3. > *IbWRKY24*

ATGCAAGTATCAGACAGAGAGCCGCCGTTCAATGGCAATGAAGATAATAATAATGACGACGAGCGCCGCGGTGATGATGATACCGTGAAGGTTGCTCGAGAAAAAATTGACTGTGAAACTAATAATAATAATAGTAATAGTAATAATAATAATGGTGCAGCGGGGGATTCCAAGCCCCTTGATAAGGCGGTGGCGGACCGCCCTCGGCGGCCGAGCATTGCCGAGAGGAGAGCTGCCAAATGCGGTTTCAAAGCTTCCAGCATCCGGTGCATGGCCTCCGACTCTTCCTCCACCGCCGCGGTGGCGGCTCCCTCTCCCTCGTCTCTCCGGTCATCTTACTTCACTATTTCTCCCGGCATTAGCCCCACGGCTTTGCTAGACTCGCCGGTCATGCTCCCCAACTCTCTTGGACAACAATCTCCTACGACAGGAACAATGCAATTTCCGGCGGCGGAGGATGAAAATTTTGGGTTTAACTTAGCCGGCGTCATAGATCATAATAGTCCAGATGAGATGAAGTCCAGCATAGGTGAATCCTCGGATCCATTTCCTTCAACCTCCGCAAATGAAACGCCGCAGGTTTCATATTCTTCTCAAATGCAGATCACTCCCTCAATACCTTTCGATTTTCCGATGGAATCCTTGGATTGCGGAGTGGAGGTATCGGGTGTCGACACTAACCCTAATTCCAATTCCAATTCGAATCTTATGTTACAGTCGCCGGTTAGTGGAGAATGCATCAATAATAATGTTAATGGAGAAAGCTGCAGCTCGTATAATAACCAGGAACAGGAGTTGGATCACCGGAGAGGGGCCGCCGCGGCGCCGGCGGCGAAATCGGTAGCTAATAATAATGCGTCTGCCGCCGCCGGTGGCGGCGCCAGTTCGTCGGACGACGGGTACACGTGGAGGAAATACGGGCAGAAGCACGTGAAAGGGAGCGAGTATCCGAGAAGCTATTACAAGTGCACTCATCCCAAATGCACCATGAAGAAAAAGGTAGAGCGTTCGCCGGACGGGCAGATCACGGAGATCGTGTATAAGGGCGCGCATAATCACCCGAAGCAGGCCACGAGTCTCCGGCGATCGCCGCCGTCGTTAGGAGCGGAAAGCTGTTCGTCGGAGATGATGGGCCAAGGCGGCGGATCTTGTTTCAGAGCTCAGGCTCCGATTTGGGCAAACATTCAGCATTATGGCAGCATGCCGGAAAGACCAACATTGGCGTCGAGCAGTGATCTGACGGCGGAGATTTGCGATCCGCTATCTTCGCTGACGACGAGATCGGCGGCCGCCATGAGTGGATTTGAATCCGCAGCAACCCCTGAACCTTCATCTACACTTGCTAGTCAAGATTGCGATGATAATGAAGATGCAGTCACCCAGGGGATCTCCAATTCCCAGTTTGGTGACGATGGAGAATCCGAGCCCAAACGAAGGAGAAAAGATGGGTGGTCAACTGAGGCAAGTTTATCAACAAGATCAATCCGGGAACCCAGAGTAGTGCTCCAGATTGAGAGTGAAATTGATATTCTTGACGACGGATATCGCTGGAGAAAATACGGCCAAAAAGTTGTCAAAGGAAATCCAAATCCAAGGAGCTATTACAAGTGCACAAGTCCCGGATGTCCGGTGAGGAAGCACGTGGAGAGAGCTTCCGACGATCTGAAATCCGTAATCACCACATACGAGGGGAAGCACAACCACGACGTGCCACCAAACAAGGCGGCCGTAGTGAACTACAACAGCTACAGCGCCGCCCCGGGCACCACGGCCAGCTCAGCCATGGCGAGAGCTCCGGCTCTCGGGGGCGTGGGGGTGCAAGATAATCATCCATCCTTCCCGTTTGAACGGAAACCCATGATTGCAGGTGGCGGTGGGGACGAGCTACTAAGGCCGGAAATGCTGAACTGCTATGCCGGCGACTTCAGGTTCGTGCCATCCTCCATTTACCCGCTCAAATTCCCTCCTCCGCCGTTGCAGGGCCGGAGCAGCCCGCTGACAGCGGCCGCAGCCGCCGCCACCTACAACTACAGCCGGCCTCCGGGCCTGGTGCTGCCGGAATTCCCCATGCCATTACTCCCAATGAGCCTGCCTCCATTCCATGAACTCACCAATTTACCTCCTCTTGCCGATTTTCTGCACTTCAATGACCCTAGCACAAAAGAAGAGCACAAGGAGAATGATCCCCACACATCATTACTATACGAATGA

4. > *IbWRKY21*

ATGGAAGAGATTGAAGAAGCTAACAGGGCAGCTGTGGAGAGTTGCCATAGGGTTATTAGTCTGTTGTCTCAGCCTCATGATCAGAGCCAGTATGCCAAATTAGCCCTAGAAACTGGGGAGGCTGTTCATAAGTTCAAGAGGGTTGTTTCTAAGCTCAATTCCACTTTGGGGCATGCAAGGGTGAGAAAGGTGAAGAAAATCCAGACCCCCTCTCTCCCTCCAAGCATTCTTTTGGAGAACCCAATGTGCAGAGGGGATGATCATCACCCCAAAGCCTTGCAGCTTCTGCCTGCCATTTCTCTTGAGGCCTCAAACCAGGAAAAGGGTTCTTCTGGTGTTAAGAGTGGCCTTGCATTGGGGAACCCTTCGTTCGAGTTGAACTTACATGGTAAAACCCCGGTTCCGTTATCCCACCAGACTCCGATTCCAAGCTATCACTTCCTTCAACAGCAGCAGCAGAGGTATCAGCAGCAGCAGCAGCAGCAATTGAAGCAACAGGCAGAGATGATTTATCGTCGTAGCAATAGTGGCATTAGTCTGAATTTCGATAGCTCTACTTGCACCCCTACCATGTCCTCCACTCGCTCGTTTATCTCCTCTCTGAGTATCGATGGGAGTGTCGCGAATATGGACGGGAATGCCTTCCATTTAATCGGTGCCTCTCGCTCCGCTGATCTGAGCTCGTATCAGCACAAGAAAAGGTGCTCTGGAAGGGGCGAGGATGGAAGTACGAAATGTGGAAGCAGCAGTCGATGCCACTGCTCCAAGAAGAGGAAACATCGGGTGAAAAGGTCAATTAAAGTTCCCGCTATCAGTAACAAGCTAGCTGATATCCCTCAAGACGAATATTCTTGGAGGAAGTACGGGCAGAAGCCAATCAAAGGTTCTCCTCACCCAAGGGGATACTATAAATGTAGTAGCATGAGAGGCTGCCCTGCGAGGAAGCATGTGGAAAGATGCTTGGAAGACCCTTCAATGTTAATTGTGACTTACGAGGGAGACCATAACCATCCTAGGGTGCCATCGCAGTCAGCAAACACATAA
