# Supplementary material for: Comparative transcriptome analysis of purple-fleshed sweet potato and its yellow-fleshed mutant provides insight into the transcription factors involved in anthocyanin biosynthesis in tuberous root
Source: Front Plant Sci. 2022 Aug 8;13:924379. doi: 10.3389/fpls.2022.924379 (PMC9393619; doi:10.3389/fpls.2022.924379)
Supplement: Supplementary Table 5 — Protein sequences used in Figure 4 and Supplementary Figure 2. [file Table_5.DOCX]

WRKY Cluster

>AtWRKY33

MAASFLTMDNSRTRQNMNGSANWSQQSGRTSTSSLEDLEIPKFRSFAPSSISISPSLVSPSTCFSPSLFLDSPAFVSSSANVLASPTTGALITNVTNQKGINEGDKSNNNNFNLFDFSFHTQSSGVSAPTTTTTTTTTTTTTNSSIFQSQEQQKKNQSEQWSQTETRPNNQAVSYNGREQRKGEDGYNWRKYGQKQVKGSENPRSYYKCTFPNCPTKKKVERSLEGQITEIVYKGSHNHPKPQSTRRSSSSSSTFHSAVYNASLDHNRQASSDQPNSNNSFHQSDSFGMQQEDNTTSDSVGDDEFEQGSSIVSRDEEDCGSEPEAKRWKGDNETNGGNGGGSKTVREPRIVVQTTSDIDILDDGYRWRKYGQKVVKGNPNPRSYYKCTTIGCPVRKHVERASHDMRAVITTYEGKHNHDVPAARGSGYATNRAPQDSSSVPIRPAAIAGHSNYTTSSQAPYTLQMLHNNNTNTGPFGYAMNNNNNNSNLQTQQNFVGGGFSRAKEEPNEETSFFDSFMP

>AtWRKY4

MSEKEEAPSTSKSTGAPSRPTLSLPPRPFSEMFFNGGVGFSPGPMTLVSNMFPDSDEFRSFSQLLAGAMSSPATAAAAAAAATASDYQRLGEGTNSSSGDVDPRFKQNRPTGLMISQSQSPSMFTVPPGLSPAMLLDSPSFLGLFSPVQGSYGMTHQQALAQVTAQAVQANANMQPQTEYPPPSQVQSFSSGQAQIPTSAPLPAQRETSDVTIIEHRSQQPLNVDKPADDGYNWRKYGQKQVKGSEFPRSYYKCTNPGCPVKKKVERSLDGQVTEIIYKGQHNHEPPQNTKRGNKDNTANINGSSINNNRGSSELGASQFQTNSSNKTKREQHEAVSQATTTEHLSEASDGEEVGNGETDVREKDENEPDPKRRSTEVRISEPAPAASHRTVTEPRIIVQTTSEVDLLDDGYRWRKYGQKVVKGNPYPRSYYKCTTPGCGVRKHVERAATDPKAVVTTYEGKHNHDLPAAKSSSHAAAAAQLRPDNRPGGLANLNQQQQQQPVARLRLKEEQTT

>PhPH3

MEVNEAAKIAIARPVASRPRCPIYRSFSELLAGAINTSSTNVHSEMGITAIKPKTVRLKPAANYALIGELSSQVGMSGAPVDCRSDNILQSAEKPKVLYKPMAKLAPRKNISLLENKGSYAPDQKREIAEDEAEGHVQSASEVKQQNGLTTESRQSLLAKSRQDKRIMQSAIVSENTEEVEQSLLNTNNVDRPSYDGYNWRKYGQKQVKGSEYPRSYYKCTHLKCPVKKKVERSHDGQIAEIVYRGEHNHPKPQPPKRNFSDGQGRALVSNDTSKETINPALSNQYPHTREANVHRIENQADVGLSTQTAYCSKPPCFYDPTSGAGMYRAFRNSEDSAERDKKLEADCDEPKTKRRKIEGQPNGAGTSGESALPHMSIQNTTDSEITEDGFRWRKYGQKVVKGNSYPRSYYRCTSPKCNVRKFVERTIDDPNALITTYEGKHNHGIPSRRPNSEASKTSSKSSAMKDKS

>VvWRKY26

MEIKESERIVVAKPVASRPTCSSFRSFSELLAGAINASPPTPCPETSFAAIRPKTVRFKPVANRAPIGVVSSQAQVSGTEVCNSSDKVLKSDSKPTVVYKPLAKLVSKTTVSLLANLGSSNMSHQQTLAQVEARVQPPNQDRQHSRPHLSSNLHQTFPSQEETDRTSEPSKTASQNLEEDQKPLLPSSNGDRPSYDGYNWRKYGQKQVKGSEYPRSYYKCTYPSCPVKKKVERSLDGQIAEIVYKGEHNHSKPQPPKRNSSGTLGQGFVSDGTGQDTNNPAWGTRLNERNEGSEGRIENQNEVGLSTHSTYPGKAPLNYDSGTAGALKAGGGTPDNSCGLSGDCEEGSKGLEPEEDEPRSKRRKSENQSSETVIVGEGAQEPRIVVQNSTDSEILGDGFRWRKYGQKVVKGNSYPRSYYRCTSLKCNVRKHVERASEDPGSFITTYEGKHNHDMPTRNTNAATSEPDMQAHTNKEKP

>FvWRKY44

MDIKEAERVVIAKPVATRPTCSSFKSFTELLTGAIDAAPSNVSSETAVPAIRPKTVRFKPTANHPVAALVSSQAETSGAAISNSAEKVSKSDSKSTVVYKPLAKVVSRATVSALANLGNFNTSQQQTQSSVGTGVVLRPNRDKSYKTQLVSNIYQKSRSCAETSQTTEPVKIVSQNMEEDAKNIPAAANSDRPSYDGYNWRKYGQKQVKGSEYPRSYYKCTHPNCPVKKKVERSLDGQIAEIVYKGEHNHSKPQPPKRSSSGTQGSGFASDATGQDYNTRLWNSHLNEKNEGSEGRVEDQNEVGIPVHSYQSKNIVHYDPLATGKLNAGTATPDNSCGVSGECEEGSKGVETEDYEPRSKRRKSENQSSEAGISGEVMQEPRVVVQSSADTEITGDGFRWRKYGQKVVKGNPYPRSYYRCTSVKCSVRKHVERVSEDPKAFITTYEGKHNHDMPLRTANPGASSEKDPQAPPSSKEKP

>AtWRKY75

MEGYDNGSLYAPFLSLKSHSKPELHQGEEESSKVRSEGCSKSVESSKKKGKKQRYAFQTRSQVDILDDGYRWRKYGQKAVKNNKFPRSYYRCTYGGCNVKKQVQRLTVDQEVVVTTYEGVHSHPIEKSTENFEHILTQMQIYSSF

>PbWRKY75

MENYPTFFSSSTAPAPFPLSLNMGNPAHHVYNSNDLHQFQNSKSSNGFLGLMSEMEASNNMIKNNSSSQEKSFGGSERSEATVRLGMKKGDQKKIRKPRYAFQTRSQVDILDDGYRWRKYGQKAVKNNKFPRSYYRCTHQGCNVKKQVQRLTKDEGVVVTTYEGMHSHPIEKSTDNFEHILSQMKIYTPF

>MdWRKY11

MAIDLVGFSKMDDRTAVQEAASAGLQSMQHLIRTLSNQTPSHTPLDCREISDFTVTKFKHLISVLNRTGHARFRRGPAKPASDSVHPKPQTTLTAFQTPKSDKDYSTTVSPPVSTTSSFFSSNTIGDGSVSNGKTFSSISVPTPPAFSAGKPPLPQSHRKRCHEGEPAKTSSSSGHCHCSKRRKSKVKRTIRVAAISSKTADIPADEFAWRKYGQKPIKGSPHPRGYYRCSTVRGCPARKHVERAQDDPKMLVVTYEAEHRHPHPYPSLTAANVGLVFQSS

>AtWRKY53

MEGRDMLSWEQKTLLSELINGFDAAKKLQARLREAPSPSSSFSSPATAVAETNEILVKQIVSSYERSLLLLNWSSSPSVQLIPTPVTVVPVANPGSVPESPASINGSPRSEEFADGGGSSESHHRQDYIFNSKKRKMLPKWSEKVRISPERGLEGPQDDVFSWRKYGQKDILGAKFPRSYYRCTHRSTQNCWATKQVQRSDGDATVFEVTYRGTHTCSQAITRTPPLASPEKRQDTRVKPAITQKPKDILESLKSNLTVRTDGLDDGKDVFSFPDTPPFYNYGTINGEFGHVESSPIFDVVDWFNPTVEIDTTFPAFLHESIYY

>AtWRKY6

MDRGWSGLTLDSSSLDLLNPNRISHKNHRRFSNPLAMSRIDEEDDQKTRISTNGSEFRFPVSLSGIRDREDEDFSSGVAGDNDREVPGEVDFFSDKKSRVCREDDEGFRVKKEEQDDRTDVNTGLNLRTTGNTKSDESMIDDGESSEMEDKRAKNELVKLQDELKKMTMDNQKLRELLTQVSNSYTSLQMHLVSLMQQQQQQNNKVIEAAEKPEETIVPRQFIDLGPTRAVGEAEDVSNSSSEDRTRSGGSSAAERRSNGKRLGREESPETESNKIQKVNSTTPTTFDQTAEATMRKARVSVRARSEAPMISDGCQWRKYGQKMAKGNPCPRAYYRCTMATGCPVRKQVQRCAEDRSILITTYEGNHNHPLPPAAVAMASTTTAAANMLLSGSMSSHDGMMNPTNLLARAVLPCSTSMATISASAPFPTVTLDLTHSPPPPNGSNPSSSAATNNNHNSLMQRPQQQQQQMTNLPPGMLPHVIGQALYNQSKFSGLQFSGGSPSTAAFSQSHAVADTITALTADPNFTAALAAVISSMINGTNHHDGEGNNKNQ

>AtWRKY40

MDQYSSSLVDTSLDLTIGVTRMRVEEDPPTSALVEELNRVSAENKKLSEMLTLMCDNYNVLRKQLMEYVNKSNITERDQISPPKKRKSPAREDAFSCAVIGGVSESSSTDQDEYLCKKQREETVVKEKVSRVYYKTEASDTTLVVKDGYQWRKYGQKVTRDNPSPRAYFKCACAPSCSVKKKVQRSVEDQSVLVATYEGEHNHPMPSQIDSNNGLNRHISHGGSASTPVAANRRSSLTVPVTTVDMIESKKVTSPTSRIDFPQVQKLLVEQMASSLTKDPNFTAALAAAVTGKLYQQNHTEK

>MdWRKY40

MDHSAAYDDDTTLDLNSRPLRLFDDTPMIKQEVHSKISIDFGRQLSSTEESGRLLEELHRVSAENKKLTEMLTVMGENYNALRSQLLEYMSKNPEKELSPISKKRKSESSNNNTNSNNIMNGAVNGNSESSSSDGESFKKPREETIKAKVSRIYVRTEASDTTSLVVKDGYQWRKYGQKVTRDNPCPRAYFKCSFAPSCPVKKKVQRSVEDQSILVGTYEGEHNHPNPSQIEATSGSNRCMTIGSVPYSTSLGSSGPTITLDLTKSKSSTADAEGTKTRTETPEVRKFLVEQMASSLTKDPDFTKALATAISGRILQHNTY

>PdWRKY40

MDYSAAYDDDTSLDLNSMPLRLFNDTPMIKKEVHSKILIDFGRQLSPKEESGALLEELHRVSAENKKLTEMLTVMGENYNALRNQLLEYMSKNPEKELSPISKKRKSESSNNNTNSNNIMNGAVNRNSESSSSDGESYKKPREEIVKAKISRACVQTEASDTSLVVKDGYQWRKYGQKVTRDNPCPRAYFKCSFAPSCPVKKKVQRSVEDQSILVATYEGEHNHPNPSQIEATSGSNNCVAIGSVPCSTSLGSTGPTITLDLTKSKSSTADAKSTKTRTETPEVRKFLVQQMASSLTKDPDFTKALAAAISGRILQHNTY

> Cluster-13485.74238(IbWRKY21)

MEEIEEANRAAVESCHRVISLLSQPHDQSQYAKLALETGEAVHKFKRVVSKLNSTLGHARVRKVKKIQTPSLPPSILLENPMCRGDDHHPKALQLLPAISLEASNQEKGSSGVKSGLALGNPSFELNLHGKTPVPLSHQTPIPSYHFLQQQQQRYQQQQQQQLKQQAEMIYRRSNSGISLNFDSSTCTPTMSSTRSFISSLSIDGSVANMDGNAFHLIGASRSADLSSYQHKKRCSGRGEDGSTKCGSSSRCHCSKKRKHRVKRSIKVPAISNKLADIPQDEYSWRKYGQKPIKGSPHPRGYYKCSSMRGCPARKHVERCLEDPSMLIVTYEGDHNHPRVPSQSANT

> Cluster-11869.0(IbWRKY24)

MQVSDREPPFNGNEDNNNDDERRGDDDTVKVAREKIDCETNNNNSNSNNNNGAAGDSKPLDKAVADRPRRPSIAERRAAKCGFKASSIRCMASDSSSTAAVAAPSPSSLRSSYFTISPGISPTALLDSPVMLPNSLGQQSPTTGTMQFPAAEDENFGFNLAGVIDHNSPDEMKSSIGESSDPFPSTSANETPQVSYSSQMQITPSIPFDFPMESLDCGVEVSGVDTNPNSNSNSNLMLQSPVSGECINNNVNGESCSSYNNQEQELDHRRGAAAAPAAKSVANNNASAAAGGGASSSDDGYTWRKYGQKHVKGSEYPRSYYKCTHPKCTMKKKVERSPDGQITEIVYKGAHNHPKQATSLRRSPPSLGAESCSSEMMGQGGGSCFRAQAPIWANIQHYGSMPERPTLASSSDLTAEICDPLSSLTTRSAAAMSGFESAATPEPSSTLASQDCDDNEDAVTQGISNSQFGDDGESEPKRRRKDGWSTEASLSTRSIREPRVVLQIESEIDILDDGYRWRKYGQKVVKGNPNPRSYYKCTSPGCPVRKHVERASDDLKSVITTYEGKHNHDVPPNKAAVVNYNSYSAAPGTTASSAMARAPALGGVGVQDNHPSFPFERKPMIAGGGGDELLRPEMLNCYAGDFRFVPSSIYPLKFPPPPLQGRSSPLTAAAAAATYNYSRPPGLVLPEFPMPLLPMSLPPFHELTNLPPLADFLHFNDPSTKEEHKENDPHTSLLYE

> Cluster-13485.101021(IbWRKY44)

MEIKEVKKIAVAKPVATRPTCSAYTPFSELLSGAINGSSTGGCSQTAVIAAIRPKTVRLRSAGNQTLGGKVEMPGVSAWSPPANVLKSDDKPTIVYKPMPKLLSKATFPQNLNMRSSTSSQQNEAAEETNQVRPSEIRLEAHQSLSLKSGTEKKPVENSKMALQNIEEDERSVFQGSGVDCLSSDGYNWRKYGQKQVKGSEFPRSYYKCTHPKCPVKKKVERSSLDDQIAEIVYKGEHNHPKPQLPRSNLRDGQPKGVLVSEDTCNETNNPVRSEQLTLQNEPCGLSTEHKNTTMLSTRSTYSSGPPPPCYPVTSAAAFHGAVSTPENSCTPSGIHREGLEAEGDELKGKRRKCGSQTNNGATLGNGAMETQTVVGSTTDSETTGDGFRWRKYGQKVVKGNTYPRSYYRCTSPKCNVRKYVERAPDDPKSFITTYEGKHNHDIPTRNPNPEASRSSTRAAATKEKS*

>PyWRKY26

MASSSGSLETSANSHPAAFTFSTHPFMTNSFSDLLEAGTDEYSNPPRYIGQGGLSDRIAERTGSGVPKFKSLPPPSLPISPPSISPSSYFAIPPGLSPAELLDSPVLLNASNILPSPTTGSFAARGAFNWKNNQQNVKQESKNHSDFSFQTQARPPISSSSSMFQSSNTSIQTTQEQAWNSQELPKQEYGSVQTLSSELTTKTLQSNAPANGGFNQQSQTLSRKSDDGFNWRKYGQKQVKGSENPRSYYKCTYPNCPTKKKVERSLDGQITEIVYKGNHNHPKPQNPRRSSSNSHAIHAFNPTNTNEIPDQTYANHGNSQMDSIGTPEHSSISIGDDDFEQSSQRSKSGGGEEFDEDEPNAKRWKNEADRNEEISAPGSRTVREPRVVVQTTSDIDILDDGYRWRKYGQKVVKGNPNPRSYYKCTNPGCPVRKHVERASHDLRAVITTYEGKHNHDVPAARGSGRPXXXXTHDVPAARGSGSHAAVNRPIPNNNNNLASAMRPITHHTNNSANTNSLQNLRQPTSEGQAPFSLEMLQSPGSYGFVGFDGNSLGSYMNQTQLSDIFSKAKEEPRDDAFFESLLC

>PyWRKY31

MDKGWGLTLDSDSFGYLLNKPPTAAVKLDHHNNKRSSFFRGERMFPGMEFPVKLGRREEQDASQPSNDNNRKVVDEVDFFSDRKNKLNTNSTDDHHQDSKSNGAVSVKKENWTGLDVNTGLQLVTANTRSDQSMVDDGISSDHGVNKTAKNHELAQLQVEVQRMNAENQRLKEMLGQVTNNYSVLQMHLAAVVQQQNHTTAAPADQRSQLDRDQNAEAKSDQEKKQGLVPRQFLNLGPRATAETNDDQVSNCSSEARTRSASPPQNIDEVVKNDQIGPLDPPNSNNFRDGKRVGGDESPESESQGWVPNKVPKLNNSAAPKPIDQSTEATMRKARVSVRARSEAPMITDGCQWRKYGQKMAKGNPCPRAYYRCTMAVGCPVRKQVQRCAEDRTILITTYEGNHNHPLPPAAMAMASTTTAAASMLLSGSMASADGMNMNPNNLLARAILPCSSSVATISASAPFPTVTLDLTTSPLQFQRPNTPFQVPNFPGQPQSFGAASQQLPQGFGQALYNQSKFSGLQLSQDLMGSTSSQQQQQMQQTQSSSFADTVSAIAADPSFTAALAAAITSIIGGGAHPSNNNSASTTSNNSSGANNNKNISSFPGN

>StWRKY13

MEVNETAKIAIVRPVASRPRCPIYKSFSELLAGAVDISSTNVHSEMAITAIRPKTVRLKPVTNHALVGERSSQVAMSEAPVGCRSDYILQSVEKPKVLYKPIAKLAPRKTIPLLENKGSSVSDQRREKAETKAGVQSANEVKQHRDLTTESKRSLLAKSGEDKKIVGSTIVSESTEEVPQSLINTSNVDRPSYDGYNWRKYGQKQVKGSEYPRSYYKCTHLKCPVKKKVERSYDAQIAEIVYRGEHNHPKPQPPKRNLSDVHVRAAVCNDTSKETNNPAWSNQHPQTSEAYVYRIENQNDFGLTIHSAHSSKAPCFYDPIAAAGMLTAVGNSEDSAEGSKRLETTCDEPKTKRRKLKGQCNRAGTSGESTFPYIPNQSTTDSEITDDGFRWRKYGQKVVKGSSYPRSYYRCTSPKCSMRKFVERTMDDPKAFITTYEGKHNHVVPNRRPNSEASKTSSKSSAMKEKS

MYB Cluster

>AtMYB90/PAP2

MEGSSKGLRKGAWTAEEDSLLRLCIDKYGEGKWHQVPLRAGLNRCRKSCRLRWLNYLKPSIKRGRLSNDEVDLLLRLHKLLGNRWSLIAGRLPGRTANDVKNYWNTHLSKKHESSCCKSKMKKKNIISPPTTPVQKIGVFKPRPRSFSVNNGCSHLNGLPEVDLIPSCLGLKKNNVCENSITCNKDDEKDDFVNNLMNGDNMWLENLLGENQEADAIVPEATTAEHGATLAFDVEQLWSLFDGETVELD

>AtMYB113

MGESPKGLRKGTWTTEEDILLRQCIDKYGEGKWHRVPLRTGLNRCRKSCRLRWLNYLKPSIKRGKLCSDEVDLVLRLHKLLGNRWSLIAGRLPGRTANDVKNYWNTHLSKKHDERCCKTKMINKNITSHPTSSAQKIDVLKPRPRSFSDKNSCNDVNILPKVDVVPLHLGLNNNYVCESSITCNKDEQKDKLININLLDGDNMWWESLLEADVLGPEATETAKGVTLPLDFEQIWARFDEETLELN

>AtMYB114

MEGSSKGLRKGAWTAEEDSLLRQCIGKYGEGKWHQVPLRAGLNRCRKSCRLRWLNYLKPSIKRGKFSSDEVDLLLRLHKLLGNRWSLIAGRLPGRTANDVKNYWNTHLSKKHEPCCKTKIKRINIITPPNTPAQKVDIF

>PtrMYB117

MVSSSGTRKGAWTREEDILLRDCVEKYGERRWHQVSPRAGLNRCGKSCRLRWLNYLKPGIKRGRYSEDEEDLIIKLHRLLGNRWTLIAGRLPGRTANDVKNYWNTNLRKKVVSGTREAQTKPEPKAITKANIIKPRPHKFKSSCWLGGEGIPFFNGGFQYGYDLCKPCSTSALSPSDIFEVERMWWESLLDDKEINVSSSTGCLRSGSESDQEPITSLFAEDSAPEGMRIGDVFCEQGQHCWSDLSFDAAELWNLVNT

>VvMYBA1

MESLGVRKGAWIQEEDVLLRKCIEKYGEGKWHLVPLRAGLNRCRKSCRLRWLNYLKPDIKRGEFALDEVDLMIRLHNLLGNRWSLIAGRLPGRTANDVKNYWHSHHFKKEVQFQEEGRDKPQTHSKTKAIKPHPHKFSKALPRFELKTTAVDTFDTQVSTSRKPSSTSPQPNDDIIWWESLLAEHAQMDQETDFSASGEMLIASLRTEETATQKKGPMDGMIEQIQGGEGDFPFDVGFWDTPNTQVNHLI

>PhAN2

MSTSNASTSGVRKGAWTEEEDLLLRECIDKYGEGKWHLVPVRAGLNRCRKSCRLRWLNYLRPHIKRGDFSLDEVDLILRLHKLLGNRWSLIAGRLPGRTANDVKNYWNTHLRKKLIAPHDQKQESKNKAVKITENNIIKPRPRTFSRPAMNNFPCWNGKSCNKNTIDKNEGDTEIIKFSDEKQKPEESIDDGLQWWANLLANNIEIEELVSCNSPTLLHEETAPSVNAESSLTQGGGSGLSDFSVDIDDIWDLVS

>PhPHZ

MNTTIPKSSGLVRKGAWTEEEDVLLRKCIEKFGEGKWHQVPVRAGLNRCRKSCRLRWLNYLRPHIKRGDFSEDEVDLIFRLHKLLGNRWSLIAGRLPGRTANDVKNYWNTHLQRKLIAPARQEIRKCRALKITENNIVRPRPRTFSNSAQNISWCSNKSITNSTIDTDGSNNECIRINDKKPMAEVSRDDGVQWWTSLLANCNENDEPAVENMSYDKLPSLLHEEISPPMNGGISDCMQEGQSGWDDFSVDIDHLWNLLN

>PhAN4

MKTSVFTSSGVLRKGSWTEEEDILLRKCIEKYGEGKWHQVPVRAGLNRCRKSCRLRWMNYLRPHIKRGDFSPDEVDLILRLHKLLGNRWSLIAGRLPGRTANDVKNYWNTNLLRRSKFAPPQQHDRKCPKAIKTMAKNAIIRPQPRNLSKLAKNNVSTIHKDEHSKQEIIIEKPTTAEVVSRDENVEWWTNLLLDNCNGFEKAATESTSAFKNIESLLNEELLSPSINGGTYYPMQETRDMGWSDLSIDADLWELL

>PhDPL

MNTSVFTSSGVLRKGAWAEEEDILLRKCIEKYGEGKWHQVPVRAGLNRCRKSCRLRWLNYLRPHIKRGDFSPEEVDLILRLHKLLGNRWSLIAGRLPGRTANDVKNYWNTHLLRRSNFAPPPQHERKCTKAVKIMAKNVIIRPQPRNLSKLAKNNVSNYSTIHKDEHSKQKMFIEKPTAAEVVSRDNNVEWWTNLLLDNCNGFEKAAPESTSAVKNIENLLNEELLSTSINGGTNYPMQETGDMGWSDFSIDSDLWELLLQ

>IpMYB1

MVNSSARWSPRVRKGAWSEEEDDLLRKCIQKFGEGKWHLVPFRAGLNRCRKSCRLRWLNYLHPDIKRGHFSLEEADLILRLHKLLGNRWSLIAGRIPGRTANDVKNYWHSHLKKKVVSMHMASSNSSRQDNNWDDEKGKAPQIKENILFRPRPRRFFRTSLSSPALSTLTGKAKAVVYDAPPPPPPPPHQLQPQPEATSPAADLLMVFNVQQNSNSIETNLPAQTTAPSSHDGVKWWEDLLYDDSHQGLIDWTTDDDFPIDVDLLKLLDTTI

>InMYB1

MVNSSARWSPRVRKGAWSEEEDDLLRKCIQKFGEGKWHLVPFRAGLNRCRKSCRLRWLNYLHPDIKRGHFSLEEADLILRLHKLLGNRWSLIAGRIPGRTANDVKNYWHSHLKKKVVGMHMASSNSSRQDNNWDDEKGKAPQIKENILFRPRPRRFFRTSLSSPALSTLTGKAKAVAYDAPPPPHHHQLQAQPEATSPPADLLMVFNVQQNNNSMATNFPAQTTAPPSHDGVKWWDLLYDDDHQGLIDWTTDDDFPIDVDLLKLLDTTI

>IbMYB3

MADSSSSEPPSGVKKGAWTEQEDNLLRKCIHKYGEGKWHLVPVRAGLNRCRKSCRLRWLNYLRPDIKRGDFNLDEIDLIMRLHKLLGNRWSLIAGRIPGRTANDVKNLWNTRLQKKTIANNTPSSGQEKWKDKAPKTTENTAVIRPRPRRFVMNSSSRTLPITGKTTTIVTSEVVQLQGHNKPPEAAESTSAPRLIENVDPNNSIIDLPGGAETSDDLGQWLDDFLLDMEFDGDGMACMQEGQIEWCDFHIDSDLLDLLS

>IbMYB1

MVISSVWSGSSSRVRKGSWSEEEDQLLRECIQKYGEGKWHLIPLRAGLNRCRKSCRLRWLNYLRPDIKRGEFSPDEIDLILRLHRLLGNRWSLIAGRIPGRTANDVKNLWNTHLQKKVSAMASSRQDNYWKGKAPEITENTVVRPRPRRFLKASSSPTTLLTGNATMVAYDGQLQEHMTTQPETTSDLLMENVQQKNLTTTLPSALETTPHDNVKWWEDVLSDKELNEEGQICWSEFPTDIDLLSELLS

>InMYB2

MVNSSSAWPPPSSSRLMRKGAWTEEEDNLLRKCIQKYGEGKWHLVPLRAGLNRCRKSCRLRWLNYLRPDIKRGDFSVDEVDLIMRLHRLLGNRWSLIAGRIPGRTANDVKNYWNTHIQKKVFAMARMQDNWKGKAPEIRENTVVRPRPRRFLNTSLSPTSKTGKATAVTYDAQIQGHTLPQPPEAIITTSDLVMENVQLNNTIATLPSELETTTSDDRVRWWEDLLFDKEFNDDEGNACMHEGQVGWTNLPIDMDLLELLS

>IbMYB2

MVNSLSSAWPSPSGLMRKGAWTEEEDNLLRKCIQKYGEGKWHLVPLRAGLNRCRKSCRLRWLNYLRPDIKRGDFSVDEVDLIMRLHRLLGNRWSLIAGRIPGRTANDVKNYWNTHIQKKVFAMAAASSRMQDNWKGKAPEISKNTVVKPQPRRFLNTSSISRTSITGKATAVTYDAQIQAHTLPQPETTTTSDLVMENVQKNDTIASFPSELETTTFDDRVQWWEELLFDKELNDEGTACMHEGQVGWSHLPTDIDLLELLS

>InMYB3

MANSSAWSGVRKGAWSEEEDNLLRKCIQEYGEGKWHLIPIRAGLNRCRKSCRLRWLNYLRPDIKRGDFKLDEVDLIMRLHKLLGNRWSLIAGRIPGRTANDVKNYWNTHIQKKVFAMASSMQDNWKGKAPEMRENNVVRPRPRRLFLNTTSSLLSGTPPLTGKATAVTFDAQIQGHNKIPQPEATSELVTKNLQENNTIITASELETTTSNDRVQWWEDFLFDNAGSTCMNQGQVDWPNFPTDMDLSELLS

>FaMYB10

MEGFGVRKGAWTKEEDELLKQFIEIHGEGKWHHVPLKSGLNRCRKSCRLRWLNYLKPNIKRGEFAEDEVDLIIRLHKLLGNRWSLIAGRLPGRTANDVKNYWNTYQRKKDQKTASYAKKLKVKPRENTIAYTIVRPRPRTFIKRFNFTERYANIEHNHSEVSYTSSLPTEPPQTLQLENVTDWWKDFSEDSTESIDRTMCSGLGLEDHDFFTNFWVEDMLLSASNDLVNISYV

>MdMYB1

MEGYNENLSVRKGAWTREEDNLLRQCVEIHGEGKWNQVSYKAGLNRCRKSCRQRWLNYLKPNIKRGDFKEDEVDLIIRLHRLLGNRWSLIARRLPGRTANAVKNYWNTRLRIDSRMKTVKNKSQEMRKTNVIRPQPQKFNRSSYYLSSKEPILDHIQSAEDLSTPPQTSSSTKNGNDWWETLLEGEDTFERAAYPSIELEEELFTSFWFDDRLSPRSCANFPEGQSRSEFSFSTDLWNHSKEE

>MdMYB10

MEGYNENLSVRKGAWTREEDNLLRQCVEIHGEGKWNQVSYKAGLNRCRKSCRLRWLNYLKPNIKRGDFKEDEVDLIIRLHRLLGNRWSLIARRLPGRTANAVKNYWNTRLRIDSRMKTVKNKSQEMRETNVIRPQPQKFNRSSYYLSSKEPILDHIQSAEDLSTPPQTSSSTKNGNDWWETLLEGEDTFERAAYPSIELEEELFTSFWFDDRLSPRSCANFPEGHSRSEFSFSTDLWNHSKEE

>AtMYB4

MGRSPCCEKAHTNKGAWTKEEDERLVAYIKAHGEGCWRSLPKAAGLLRCGKSCRLRWINYLRPDLKRGNFTEEEDELIIKLHSLLGNKWSLIAGRLPGRTDNEIKNYWNTHIRRKLINRGIDPTSHRPIQESSASQDSKPTQLEPVTSNTINISFTSAPKVETFHESISFPGKSEKISMLTFKEEKDECPVQEKFPDLNLELRISLPDDV

DRLQGHGKSTTPRCFKCSLGMINGMECRCGRMRCDVVGGSSKGSDMSNGFDFLGLAKKETTSLLGFRSLEMK

>PhMYB4

MGRSPCCEKAHTNKGAWTKEEDERLIAYIKAHGEGCWRSLPKAAGLLRCGKSCRLRWINYLRPDLKRGNFTEDEDELIIKLHSLLGNKWSLIAGRLPGRTDNEIKNYWNTHIRRKLLSRGIDPTTHRIMNEPSTQKVTTISFAAGNEDIKDQKISIKAEFEQIKDDEIISKPIKEQCPDLNLELKISPPYQQHSDRALQQSTTGSGGASTICFTCSLGLKNNKGCSCSRNRSMNVAGYDFLGLKTNGLDYRTLETRTK

>MdMYB16

MGRSPCCEKAHTNKGAWTKEEDDRLIAYIRAHGEGCWRSLPKAAGLLRCGKSCRLRWINYLRPDLKRGNFTEEEDELIIKLHSLLGNKWSLIAGRLPGRTDNEIKNYWNTHIRRKLLTRGIDPTTHRPLNETPQESATTISFAAASANIKEEDKKISITNGLVCKDSKNPVQERCPDLNLDLQISPPCQPQQPSDGLKSGGRGLCFSCSLGLQDAKNCSCGRDAIGGATSGTTNIGYDFLGLKNGVLDYRSLEMK

>AtMYB32

MGRSPCCEKDHTNKGAWTKEEDDKLISYIKAHGEGCWRSLPRSAGLQRCGKSCRLRWINYLRPDLKRGNFTLEEDDLIIKLHSLLGNKWSLIATRLPGRTDNEIKNYWNTHVKRKLLRKGIDPATHRPINETKTSQDSSDSSKTEDPLVKILSFGPQLEKIANFGDERIQKRVEYSVVEERCLDLNLELRISPPWQDKLHDERNLRFGRVKYRCSACRFGFGNGKECSCNNVKCQTEDSSSSSYSSTDISSSIGYDFLGLNNTRVLDFSTLEMK

>AtMYB7

MGRSPCCEKEHMNKGAWTKEEDERLVSYIKSHGEGCWRSLPRAAGLLRCGKSCRLRWINYLRPDLKRGNFTHDEDELIIKLHSLLGNKWSLIAARLPGRTDNEIKNYWNTHIKRKLLSKGIDPATHRGINEAKISDLKKTKDQIVKDVSFVTKFEETDKSGDQKQNKYIRNGLVCKEERVVVEEKIGPDLNLELRISPPWQNQREISTCTASRFYMENDMECSSETVKCQTENSSSISYSSIDISSSNVGYDFLGLKTRILDFRSLEMK

>IbMYB4a

MGRSPCCEKAHTNKGAWTKEEDERLIAYIKAHGEGCWRSLPKAAGLLRCGKSCRLRWINYLRPDLKRGNFTHDEDELIIKLHSLLGNKWSLIAGRLPGRTDNEIKNYWNTHIRRKLLSRGIDPTTHRPINGGAEPPKETTISFGAVKPEDAENNNSITGKDSEPKKEENKEETLLFKSEEPQVAEACPDLNLELRISPPSSQETRPPPLPLEAAKSGGGGRVNGLCFACILGIPNSIDCTCNNNEDYSSGSSSSN

>AtMYB3

MGRSPCCEKAHMNKGAWTKEEDQLLVDYIRKHGEGCWRSLPRAAGLQRCGKSCRLRWMNYLRPDLKRGNFTEEEDELIIKLHSLLGNKWSLIAGRLPGRTDNEIKNYWNTHIKRKLLSRGIDPNSHRLINESVVSPSSLQNDVVETIHLDFSGPVKPEPVREEIGMVNNCESSGTTSEKDYGNEEDWVLNLELSVGPSYRYESTRKVSVVDSAESTRRWGSELFGAHESDAVCLCCRIGLFRNESCRNCRVSDVRTH

>FaMYB1

MRKPCCEKTETTKGAWSIQEDQKLIDYIQKHGEGCWNSLPKAAGLRRCGKSCRLRWINYLRPDLKRGSFGEDEEDLIIRLHKLLGNRWSLIAGRLPGRTDNEVKNYWNSHLKKKILKTGTTLRPNKPHENNHAPNNKLVKLFNKMDDEVVDEVSSADSAAGCLVPELNLDLTLSIKTSTGMADPQVA

>TrMYB133

MRKPSCDIKLEKNINKGVWSKQEDQKLIDYINKHGEVCWSTLPQAAGLLRCGKSCRLRWMNYLRPDVKRGNFGEDEEDLIIKLHALLGNRWSLIAGRLPGRTDNEVKNFWNSRIRKKLIRKGIDPNNHNLHHKIPPLQNQIMSNSLKYFGLKEISKNETTKTHLDNYDKVSNATSGNKDESYALLDLNLELSL

>TrMYB134

MRTPCCDKENINKGAWSKQEDKKLIDYIQVHGEGCWGSIPKAAGLHRCGKSCRLRWLNYLRPDIKRGIFAQDEEDLIIKLHALLGNRWALIAGRLPGRTDNEVKNYWNSHIRRKLIKMGIDPNNHKLHKGFPSPHHVFAAGTSSSSCDKERNINNLTLIKSKSCEEEYINSSVSFTKKETSIINNSSSSLNLELTIALPSPNDCESPKIRDIDIDLNC

>PtrMYB182

MRKPCCDKRGNNKGAWSTEEDQKLIDYIQTHGEGCWRSIPEAAGLHRCGKSCRLRWINYLRPDIKRGNFGQDEEDLIIKLHALLGNRWSLIAGRLPGRTDNEVKNYWNSHLKKKLIDMGIDPNNHRLNQILPRLQAEPAAPVIATSTTTGSKNNVAASKPKNLSDGDSDRVSDTATCLEDDYESLVTQQAATSGSSSINIDLNIAAPASPGHRTTFGNNQQNCKWGKTSQVERDPSSLPTLLLFR

>IbMYB27

MRKASCDHSHHHHEINKGAWSKQEDQKLLDYIRKHGEGGWRDLPKAAGLLRCSKSCRLRWMNHLKQTAKRGNFGDDEEDLIIKLHALLGDRWSLIAGRLPGRTDEEVKNYWNSHIKKKLLDMGIDPNNHRLSCTHNTAAQTSGGKSRVTSPEKQRVESDGEVSDAGSSNVR

>PhMYB27

MRKACCDNKEEMHRGAWSKQEDQKLIDYITKHGAGCWRNLPKADGLLRCGKSCRLRWMNYLSPNLKRGNFSEDEEDLIIKLHALLGNRWSLIAGRLPGRTDNEVKNYWNSHLRRKLIKMGIDPKNHRISHYLHRKRLEYWSENSSRGTDHEVVSDAGSSCAKHQPSSLPDLNSPPSIHSSCAQP

>VvMYBC2-L1

MRKPCCDKQDTNKGAWSKQEDQKLIDYIRKNGEGCWRTLPQAAGLLRCGKSCRLRWINYLRPDLKRGDFAEDEEDLIIKLHALLGNRWSLIAGRPPGRTDNEVKNYWNSHLRRKLINMGIDPNNHRLSHNFPRPRDPCTAATATSSGLNNHASPPVKSVGDNDQTSDAGSCLDDNRRALPDLNLDVAITIPQPSVDTTEEAKKHNEPKVSRELEPGPSSTLLLFG

>IbMYB4b

MGRSPCCEKAHTNKGAWTKEEDQRLINYIRSHGEGCWRSLPKAAGLLRCGKSCRLRWINYLRPDLKRGNFTEEEDDLIIKLHSLLGNKWSLIAARLPGRTDNEIKNYWNTHIKRKLLSRGLDPQTHRPINAAAAAGGGGGGSAAKDICLDFRNAAAPAKSSNEKATLSLSQEDTKCNSGTTTEESQSHQQQKDDQTALNLGLSIGLSTAAETPSSSNTAESVAPPQAPPTAAAVGYAAMTQSVCLCWQLGWSPSGKLCTKCHNSYKWFP

>IbMYB4c

MGRSPCCEKAHTNKGAWTKEEDQLLINYIRLHGEGCWRSLPKAAGLLRCGKSCRLRWINYLRPDLKRGNFTQQEDDLIIKLHSLLGNKWSLIAARLPGRTDNEIKNYWNTQIKRKLISRGIDPQTHRPLDSSAGAGAGTGTTKPENISMDLSSSAPSQEETKCSSGTTSEESNHQSLKDKQRNEQMGGLDLSIGLALHPKTEDSAESTASGELLPVAPPPPAAVELSVTEAVCLCWQLGSRTGGLCNKCHTTKCFLG

>PhMYBx

MADKGQSSSSVNTPADSQDGVAPRMLVSGKTSKVAEIKFSEEEEDLIIRMYNLVGERWSLIAGRIPGRSAEEIEKYWNTRSSTSQ

> Cluster-13485.79633 (IbMYBx-ZZ)

MADLDTNSSTCGTEAQVDSIVEVTSKDLEKELVFSEDEEILITRMFNLVGERWSLIAGRIPGRTAEEIEK

YWNSRYSTSQ

>IbMYBx

MADLDNSSTCGEACVESPEVEVTSQDSNKLVFSVDEEALIVRMYNLVGERWSLIAGRIPGRSAEEIEKYWNSTHVLN

>AtETC1

MNTQRKSKHLKTNPTIVASSSEEVSSLEWEEIAMAQEEEDLICRMYKLVGERWDLIAGRIPGRTAEEIER

FWVMKNHRRSQLR

>AtCPC

MFRSDKAEKMDKRRRRQSKAKASCSEEVSSIEWEAVKMSEEEEDLISRMYKLVGDRWELIAGRIPGRTPEEIERYWLMKHGVVFANRRRDFFRK

>PtrMYB179

MESMNRRRRRKQPKINSSESEEVSSIEWEFINMSEQEEDLIYRMHKLVGERWDLIAGRIPGRKAEEIERFWIMKHREGFAGNGKLYNEVKSRTSS

>AtTRY

MDNTDRRRRRKQHKIALHDSEEVSSIEWEFINMTEQEEDLIFRMYRLVGDRWDLIAGRVPGRQPEEIERYWIMRNSEGFADKRRQLHSSSHKHTKPHRPRFSIYPS

>AtETC2

MDNTNRLRLRRGPSLRQTKFTRSRYDSEEVSSIEWEFISMTEQEEDLISRMYRLVGNRWDLIAGRVVGRKANEIERYWIMRNSDYFSHKRRRLNISPFFSTSPLNLQENLKL
